# Efficacy of herbal medications in managing painful post-traumatic trigeminal neuropathy: a systematic review of studies in rodents

**DOI:** 10.3389/froh.2026.1754478

**Published:** 2026-01-21

**Authors:** Hajer Jasim, Akiko Shimada, Evelina Wang, Khalid Almas, Nikolaos Christidis, Fawad Javed

**Affiliations:** 1 Division of Oral Rehabilitation, Department of Dental Medicine, Karolinska Institutet, Huddinge, Sweden; 2 Department of Orofacial Pain and Jaw Function, Public Dental Services, Eastmaninstitutet, Stockholm, Sweden; 3 Department of Oral Health Sciences, Faculty of Health Sciences, Osaka Dental University, Osaka, Japan; 4 Preventive Dental Sciences Department, Division of Periodontics, Imam Abdulrahman Bin Faisal University, College of Dentistry, Dammam, Saudi Arabia; 5 Department of Orthodontics and Dentofacial Orthopedics, Eastman Institute for Oral Health, University of Rochester, Rochester, NY, United States

**Keywords:** corydalis yanhusuo, kampo, plamatine, post-traumatic trigeminal neuropathy, puerarin, uyakujunkisan

## Abstract

**Background:**

Post-traumatic trigeminal neuropathy is conventionally treated with pharmacologic medications such as carbamazepine. Studies have reported that herbal medications (HM) exhibit antinociceptive properties and are helpful in managing painful post-traumatic trigeminal neuropathy (PPTTN).

**Objective:**

The purpose is to systematically review preclinical studies that assessed the antinociceptive efficacy of HM in managing PPTTN.

**Methods:**

The focused question was “Can HM reduce PPTTN-related nociception?” A comprehensive search of indexed literature was performed up to and including November 2025. Various keywords were used in different combinations using Boolean operators: Original studies that evaluated the effects of HM on PPTTN-related nociception were included. Study protocols, reviews, editorials, case reports/series, observational studies, and letters-to-the-editor were excluded. The risk of bias (RoB) and quality of evidence were assessed.

**Results:**

Four studies on rodents were included. Three studies were performed in rats and one in mice. Protocols for experimental neuropathic pain induction, HM type and delivery and outcome variables were inconsistent in all studies. All studies reported that HM increase the induced pain thresholds in animals with induced PPTTN. Sample-size estimation was performed in none of the studies. The RoB and quality of evidence were graded as “high” and “very low”, respectively.

**Conclusion:**

The antinociceptive efficacy of HM in the management of experimentally induced PPTTN remains inconclusive, primarily due to methodological inconsistencies within the existing literature.

**Systematic Review Registration:**

https://www.crd.york.ac.uk/prospero/, identifier CRD42023486191.

## Introduction

Nociception involves a complex network of sensory neurons, neurotransmitters, and central nervous system pathways and plays an essential role in pain perception (1, 2). Dysregulation of nociception often leads to chronic pain conditions that may significantly impair the quality of life of vulnerable patients. One such condition is painful post-traumatic trigeminal neuropathy (PPTTN), a secondary orofacial neuropathic pain (NP) condition that arises after an identifiable injury to one or more trigeminal branches (3, 4). This condition is usually iatrogenic and is associated with dental extraction, endodontic therapy, and/or implant surgery (3, 5, 6). The pain is often described as a persistent or recurrent burning, aching, stabbing sensation that is localized to the affected trigeminal territory (7); and exacerbated by oral functions including speech and mastication (8). The management of PPTTN is generally multimodal and is largely adapted from established treatment approaches for NP. Commonly used pharmacologic options include gabapentinoids (such as gabapentin and pregabalin), tricyclic antidepressants, and serotonin–norepinephrine reuptake inhibitors, along with topical or local anesthetic therapies and psychological or behavioral interventions (9–12).

Herbal medications (HM) have gained attention as potential alternatives to traditional pharmacologic medications for managing pain due to their anti-inflammatory, antioxidant, and neuroprotective properties (13–17). Results from a study (18) in mice showed that *Ginkgo biloba* extracts possess anti-inflammatory and neuroprotective properties that help reduce experimentally-induced PPTTN. Likewise, another study (16) reported that *puerarin* (an active ingredient of *radix puerariae*in) effectively mitigates the expression of destructive inflammatory cytokines, including interleukin-1beta (IL-1β), within the neurons in mice; thereby offering a novel perspective on the preventive and therapeutic aspects of managing PPTTN (16). It is therefore compelling to consider HM as a potentially efficacious and robust treatment strategy for the management of pain in patients with PPTTN. A limited number of studies (16, 17, 19–22) have investigated the antinociceptive effects of HM for the management of PPTTN. However, a critical review of such investigations remains undocumented in indexed literature. The purpose is to systematically review preclinical studies that assessed the antinociceptive efficacy of HM in managing PPTTN.

## Materials and methods

### Ethical approval and PROSPERO registration

The present study is a systematic review of ethically approved and peer-reviewed indexed literature. Therefore, the study protocol was exempted from attaining prior ethical approval from an institutional review board. The present systematic review has been registered with the International Prospective Register of Systematic Reviews (PROSPERO registration ID: CRD42023486191). The literature search was done in accordance with the guidelines of the Preferred Reporting Items for Systematic Reviews and Meta-analysis (PRISMA) (23).

### Inclusion and exclusion criteria

In the present systematic review, original clinical and preclinical (animal model) studies that evaluated the antinociceptive efficacy of HM toward PPTTN were considered eligible for inclusion. The following exclusion criteria were used: study protocols, editorials, letters, legal cases, interviews, case-reports/case-series, duplicates, observational studies, and review articles. Studies based on duplicated or overlapping data, as well as those not specifically investigating the effects of HM on PPTTN-related nociception were also excluded.

### Literature search protocol

In collaboration with the librarian at the Karolinska Institutet University Library, Huddinge, Sweden, a search strategy was developed to identify both clinical and pre-clinical studies investigating whether HM reduce TN-related nociception. The electronic search was conducted up to and including November 2025 with no time or language barriers. The following databases were searched: MEDLINE, EMBASE, CINAHL, the Cochrane Central Registry of Controlled Trials (CENTRAL), Web of Science, and Scopus. The search strategies were peer-reviewed by NC before implementation. For each search concept, a medical subject heading (MeSH-terms) and free-text terms were used. The search was then translated, in part, using Polyglot Search Translator (24) into the other databases. Duplicate records were removed according to the method described elsewhere (25). Hand searches of relevant original and review articles were also conducted. Full search strategies for all databases are provided in Supplementary Appendix A.

The Rayyan tool (26) was used to assist the screening of titles and abstracts. Two authors (anonymized) independently screened the titles and abstracts. In cases of disagreement regarding the eligibility of a study, a third author (anonymized) was consulted to resolve the conflict through discussion. All potentially eligible studies were retrieved in full text. When articles were published in languages other than English, they were reviewed and, when necessary, translated by team members (anonymized) to assess eligibility and interpret the content. Any disagreements during full-text screening were again resolved through discussion with the third author (anonymized). The search was performed using a combination of the following terms: Herbal; trigeminal neuralgia; Puerarin; Uyakujunkisan; plamatine; kampo; and Corydalis yanhusuo. Boolean operators (AND, OR) were used to refine and broaden the search as needed.

### Risk of bias assessment and certainty of evidence

Two reviewers (anonymized) conducted the assessment of the risk of bias (RoB) among included studies using the Systematic Review Centre for Laboratory Animal Experimentation (SYRCLE) risk of bias tool (27). Two independent reviewers (anonymized) evaluated each study across the ten domains of the SYRCLE tool: sequence generation, baseline characteristics, allocation concealment, random housing, blinding of caregivers/investigators, random outcome assessment, blinding of outcome assessor, incomplete outcome data, selective outcome reporting, and other sources of bias. Each domain was judged as having a “low,” “high,” or “unclear” risk of bias, based on the information reported in the article. Discrepancies between reviewers (anonymized) were resolved through discussion or by consulting a third reviewer (anonymized) when consensus could not be reached.

The evaluation of evidence quality and the formulation of recommendations were conducted utilizing the Grading of Recommendations Assessment, Development, and Evaluation (GRADE) approach (28). Two independent reviewers (anonymized) assessed five domains: RoB, inconsistency, indirectness, imprecision, and publication bias. Discrepancies between reviewers (anonymized) were resolved through discussion or consultation with a third reviewer (anonymized). The certainty of evidence ratings were categorized as high, moderate, low, or very low.

## Results

### Literature search outcome

**Figure 1:**
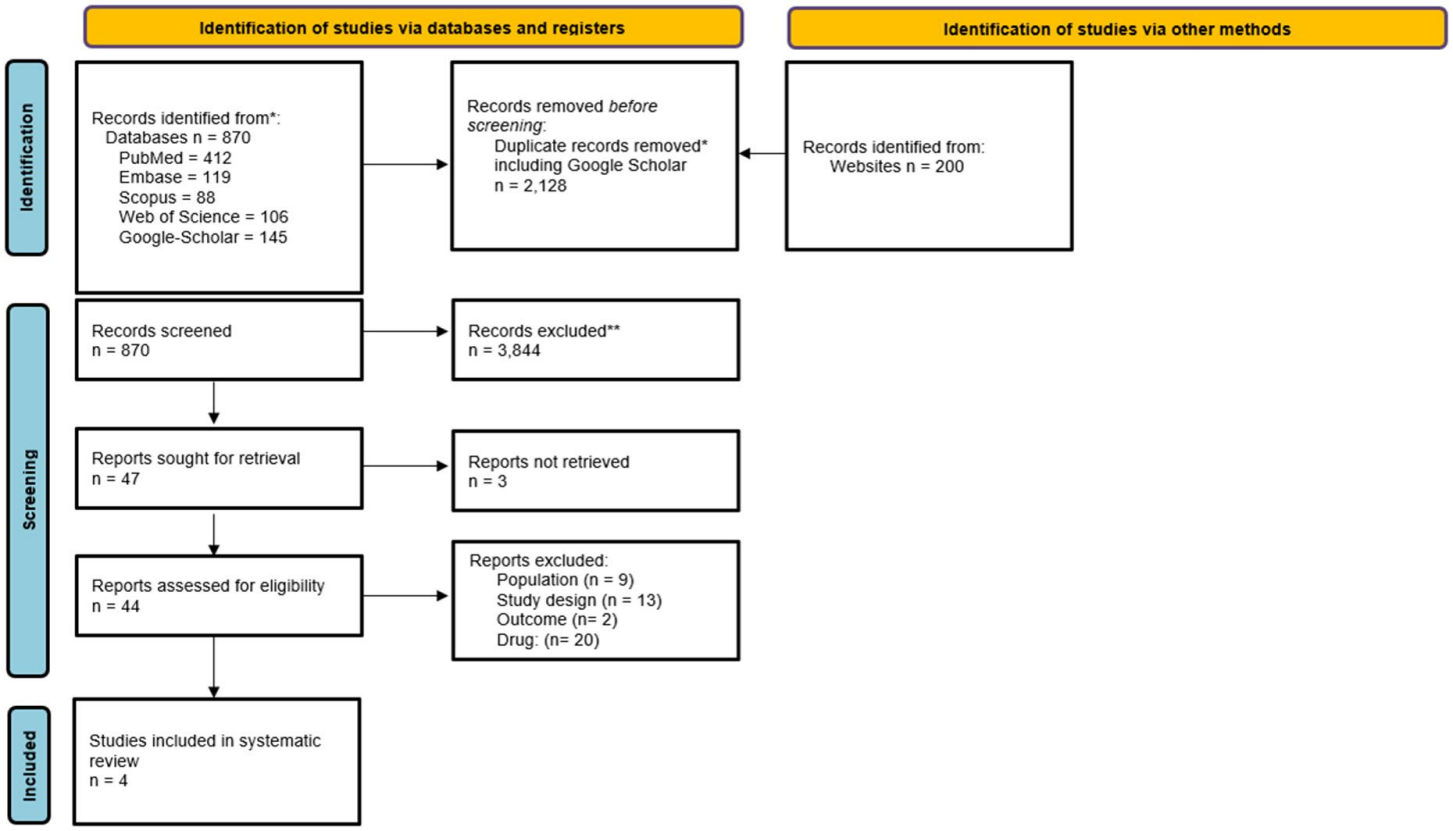
PRISMA flowchart.

The literature search protocol is shown in Appendix A. The comprehensive electronic search yielded 5,699 articles across all databases. After the removal of 2,128 duplicates, a total of 3,891 unique article titles and abstracts were screened. Of these 3,844 articles were excluded based on titles and abstract review, leaving 47 articles for full-text assessment. After assessing the full-text articles, 40 studies did not meet the inclusion criteria and were excluded, resulting in a total of four preclinical (animal-model) studies (16, 17, 19, 20) which were included in this systematic review. Figure 1 presents the PRISMA flow diagram illustrating the study selection process.

### General characteristics of included studies

The four preclinical studies (16, 17, 19, 20) were performed in rodents. Three (17, 19, 20) studies (16, 17, 19, 20) were performed in male rats (*n* = 24–46) weighing between 180 and 350 grams. The mean age of rats was reported in none of the studies (17, 19, 20). One study (16) was performed on 24 male 8-week-old mice weighing between 20 and 24 grams. The studies (16, 17, 19, 20) utilized different protocols to induce PPTTN. Du et al. (16) injected a complete Freund's adjuvant (CFA) into the unilateral facial region of mice. Liu et al. (17) and Huang et al. (20) induced PPTTN via exposure and loose ligation of the right infraorbital nerve and chronic constriction injury of the infraorbital nerve, respectively. Sunagawa et al. (19) performed ligation of the right mental nerve to induce PPTTN. None of the included studies (16, 17, 19, 20) reported performing sample-size estimation (SSE) (Table 1).

**Table 1:** General characteristics of included studies.

Authors et al.	Animal model (n)	Gender	Age (weight range)	PPTTN induction	Herbal medication (dosage)	Route of administration	SSE
Du et al. (16)	Mice (24)	Male	8-weeks (20–24 g)	CFA injection unilateral face	Puerarin (100 mg/kg/day Resveratrol (100 mg/kg/day)	Subcutaneous	No
Liu et al. (17)^*^^,^^†^	Rats (24)	Male	NR (180–220 g)	Right infraorbital nerve was exposed and loosely ligated	Palmatine (20 mg/kg a day for 2 weeks)	Intraperitoneal	No
Sunagawa et al. (19)	Rats (40)	Male	NR (250–300 g)	Right mental nerve ligation	TJ-10 (400 g/day)	Dietary	No
Huang et al. (20)	Rats (46)	Male	NR (300–350 g)	Chronic constriction injury of the infraorbital nerve	dl-THP (2 mg/Kg)	Intraperitoneal	No

CFA, complete Freund's adjuvant; dl-THP, Corydalis Yanhusou; NR, not reported; SSE, sample-size estimation; TJ-10, Saiko-Keishi-To; PPTTN, painful post-traumatic trigeminal neuropathy (PPTTN).

* Right infra-orbital nerve was exposed but not ligated.

† The facial area overlying the infraorbital nerve was stimulated to induce pain and the threshold for mechanical nociception was measured at 1, 3, 5, 7, 9, 11, and 13 days.

### Study groups, euthanasia time points, and methods of neuropathic pain assessment

The types of HM and their dosages varied across the studies included (16, 17, 19, 20). Liu et al. (17) administered Palmatine intraperitoneally (20 mg/kg daily) for two-weeks. Du et al. (16) administered Puerarin (100 mg/kg/day) and Resveratrol (100 mg/kg/day) subcutaneously to assess their anti-nociceptive effects. Sunagawa et al. (19) used a dietary regimen of Saiko-Keishi-To (TJ-10) at 400 g/day and Huang et al. delivered dl-Tetrahydropalmatine (dl-THP) intraperitoneally at 2 mg/kg. The control groups also varied among the included studies (16, 17, 19, 20). Du et al. (16) and Huang et al. (20) subcutaneously administered saline and performed sham surgery, respectively in the control groups. In the study by Sunagawa et al. (16) rats in the control group were either fed a diet lacking 0.4% TJ-10 supplementation or administered carbamazepine after PPTTN induction. Liu et al. (17) either performed sham surgery or provided no treatment to animals in the control-group after PPTTN induction. The animals were euthanized between 12 and 28 days post-intervention (16, 17, 19, 20). Pain response was assessed using mechanical stimuli, thermal, electric or pressure (16, 17, 19, 20). Three studies (16, 17, 20) performed western blotting (WB) to quantify protein expression in the trigeminal ganglion (TG). Du et al. (16) assessed the expression of Transforming Growth Factor 1-beta (TGF-*β*1) and decapentaplegic homolog3 (Smad3) proteins; and Liu et al. (17) evaluated levels of Brain-derived neurotrophic factor (BDNF) and its receptor, Tropomyosin receptor kinase B (TrkB) in the TG. Huang et al. (20) assessed the expression of Cannabinoid receptor 1 in nerves using WB. Du et al. (16) assessed cell apoptosis using the cell culture method, and Liu et al. (17) used immunohistochemical analysis to evaluate Changes in the levels of BDNF/TrkB in TG. Sunagawa et al. (16) evaluated the nerve fiber structure and inflammatory cells in the TG using histologic assessment of the TG. Du et al. (16) used polymerase chain reaction (PCR) to assess the expression of genes related to Sirtuin-1 protein and IL-1β, IL-18, and TNF-α in the TG; and Liu et al. (17) investigated the expression of pain-related genes (BDNF and TrkB) using PCR (Table 2).

**Table 2:** Study groups, euthanasia, methods and parameters assessed in included studies.

Authors et al.	Groups		Euthanasia	Methods	Parameter/s assessed
	Test-group/s	Control-group/s			
Du et al. (16)	CFA injection on unilateral face at 3 sites (30 μL/site) (*n* = 6). Subcutaneous CFA injection + Puerarin (*n* = 6). Subcutaneous CFA injection + Resveratrol (*n* = 6).	Saline treatment (*n* = 6).	After 12 days	Electric stimulus Cell culture Western blot PCR	Head withdrawal Cell apoptosis Expression of TGF-*β*1 and Smad3 proteins in the TG. mRNA expression of Sirtuin-1 protein and IL-1β, IL-18, and TNF-α in the TG.
Liu et al. (17)	Sham+10% intraperitoneal palmatine injection (*n* = 6). Induced PPTTN+10% intraperitoneal palmatine injection (*n* = 6).	Sham treatment (*n* = 6). PPTTN alone (*n* = 6).	After 14 days	Mechanical allodynia Immunohistochemical assessment Western blotting PCR	Response to simulation: rapid retreat, curling up to cage wall, hiding face and/or head under body, aggressive behavior e.g., biting, and face scratching at least three times. Changes in the levels of BDNF/TrkB in TG. BDNF and TrkB protein levels in TG Expression of pain-related genes (BDNF mRNA, TrkB mRNA and NF-*κ*B mRNA)
Sunagawa et al. (19)	Diet with 0.4% TJ-10 14 days after PPTTN induction (*n* = 20).	Diet without 0.4% TJ-10 14 days after PPTTN induction (*n* = 12). Treatment with carbamazepine after PPTTN induction (*n* = 8).	After 28 days	Pressure and thermal stimuli in the lower lip. Histologic assessment of nerve cells in the TG	Pain response (escape reaction). Assessment of inflammatory cells, preservation of nerve fiber structure and swelling in nerve cells.
Huang et al. (20)	dl-THP injection after surgery (*n* = 34)	Sham surgery (*n* = 6).	After 14 days	Pressure Western blot	Reduction in pain behavior Expression of CBI receptors in nerve

BDNF: Brain-derived neurotrophic factor dl-THP: Corydalis Yanhusou IL: Interleukin PPTTN: painful post-traumatic trigeminal neuropathy TG: Trigeminal ganglion TrkB: Tropomyosin receptor kinase B Smad3: decapentaplegic homolog3 TGF-β1: Transforming growth factor 1-beta TNF-α: Tumor necrosis factor-alpha CBI-1: Cannabinoid receptor 1.

### Outcomes

All included studies (16, 17, 19, 20) showed that an increased threshold to pain stimulus following HM. Du et al. (16) reported that HM enhance the expression of TGF-*β*1 and Smad3 in the TG. Additionally, the results demonstrated that HM decreased the expression of pro-inflammatory cytokines (IL-1β, IL-18, and TNF-α), and reduced cellular apoptosis compared to controls (16). Liu et al. (17) reported that HM downregulated the BDNF protein and its receptor (TrkB) in the TG. Histopathological results by Sunagawa et al. (19) showed reduced infiltration of inflammatory cells and preservation of nerve fiber structure in rats fed with a diet containing TJ-10 (Table 3).

**Table 3:** Outcomes of included studies.

Authors et al.	Outcome parameters			
	Response to pain stimulus	Histologic outcomes	Western blotting	PCR
Du et al. (16)	Head withdrawal threshold increased after treatment with Puerarin and Resveratrol.	Puerarin inhibited neuronal cell apoptosis.	Puerarin hindered TGF-β1/Smad3 signaling.	Puerarin and Resveratrol increased the expression of sirtuin-1 mRNA protein. Puerarin inhibited mRNA expression of IL-1β and TNF-α.
Liu et al. (17)	Palmatine reduced pain behavior by reducing the response to facial stimulation.	Palmatine significantly reduced the amount of BDNF and TrkB staining in the TG tissues.	Palmatine significantly reduced the levels of BDNF and its receptor (TrkB) in the TG tissues.	Palmatine (50 mg/kg) significantly reduced the levels of BDNF mRNA, TrkB mRNA and NF-κB mRNA
Sunagawa et al. (19)*	Pain threshold was higher in TJ-10-treated (84.7%) than carbamazepine-treated rats (67.2%).	Fewer inflammatory cells, better preservation of nerve fiber structure and less swelling and degeneration of nerve cells.	NA	NA
Huang et al. (20)	Pain threshold was higher in dl-THP-treated than untreated rats.	NA	Corydalis yanhusuo increased the expression of CB1 receptors in the inferior orbital branch of the TN.	NA

NA. not assessed; IL-1β, interleukin 1-beta; TG, trigeminal ganglion; TN, trigeminal nerve; TNF-α, tumor necrosis factor alpha.

* Unlike CBZ, TJ-10 did not cause drowsiness or movement problems in rats.

### Risk of bias and GRADE analyses

**Figure 2:**
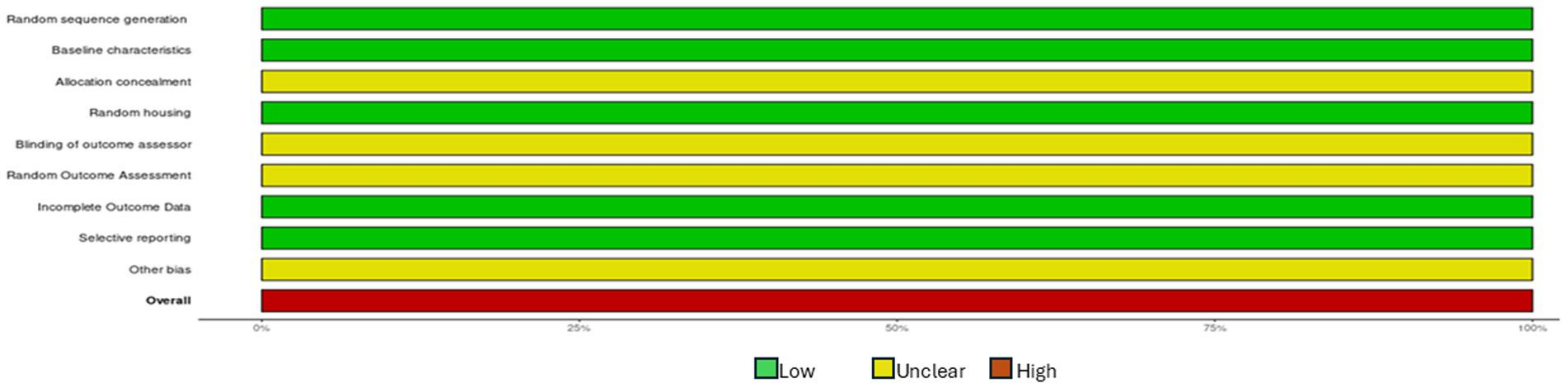
Assessment of the risk of bias within studies using the SYRCLE tool (27).

All studies (16, 17, 19, 20) had a high RoB (Figure 2). The certainty of evidence was graded as “very low” across all studies (16, 17, 19, 20) (Table 4).

**Table 4:** GRADE analysis.

Outcome	Number of studies	Study design	Risk of bias	Inconsistency	Indirectness	Imprecision	Publication bias	Overall certainty
Pain threshold for induced neuropathic pain with herbal medications	4	Experimental (animal-model) studies	Serious No sample-size calculation, variable methods	Not serious All studies showed a similar direction of effect	Serious Rodent models, limited direct application to humans	Serious No sample-size calculation, variable methods	Undetected Possibly due to a limited number of studies.	⊕○○○ Very low

## Discussion

### Identification and summary of relevant preclinical studies

A stringent literature search revealed four studies (16, 17, 19, 20) on rodent models that addressed the predefined PICO criteria and research question. A consistent result from all preclinical studies was that HM are effective in attenuating nociceptive responses in rodents with experimentally induced PPTTN. Considering these findings, it is tempting to suggest that HM are a viable alternative to conventional pharmacological agents (such as carbamazepine and tricyclic antidepressants) for the management of PPTTN-related pain. However, such a conclusion must be approached with caution, as multiple confounding factors appear to have influenced the outcomes of the included preclinical studies (16, 17, 19, 20).

### Heterogeneity in herbal medicines and their methodologies and variability in outcome measures

A notable challenge in synthesizing the findings of the included studies (16, 17, 19, 20) was the heterogeneity in the type of HM investigated, their dosages, and routes of administration. For instance, Du et al. (16) evaluated the antinociceptive effects of subcutaneously administered Puerarin and Resveratrol, each at a dosage of 100 mg/kg/day; whereas, Liu et al. (17) investigated the effects of Palmatine administered intraperitoneally at 20 mg/kg/day for the management of NP. Meanwhile, Sunagawa et al. (19) employed a different approach by administering Saiko-Keishi-To, a traditional Japanese Kampo herbal formulation, through dietary supplementation and assessing nociceptive outcomes. Another factor that complicated the interpretation of results was the heterogeneity in secondary outcome measures among the included studies (16, 17, 19, 20). For instance, some studies (16, 17, 19) conducted histological or immunohistochemical analyses of cellular changes within the TG; while others (17, 20) focused on the expression of inflammatory proteins in supernatants of TG cells. These variations in experimental design make it difficult to perform direct comparisons or determine the type and dosage of HM that may be most effective in managing PPTTN. The authors perceive that such variations complicate the extrapolation of preclinical outcomes to human populations, where pharmacokinetics, safety profiles, and therapeutic responses may differ considerably.

### Sample-size estimation

It is well-established that prior sample-size estimation **(**SSE) is a critical component of a well-designed study protocol (29). It ensures that the study is adequately powered to detect statistically significant differences (should one exist) among the groups being compared, and reduces the risk of both Type-I errors and Type-II errors (30). A critical assessment of the methodology revealed that prior SSE had not been performed in any of the preclinical studies included in the present systematic review. Therefore, it was difficult to discern whether observed differences in pain thresholds in animals treated with or without herbal supplements reflected true effects or are merely the result of random variation.

### Clinical perspectives

From a clinical perspective, the literature search involving screening of over 3,800 articles showed one RCT (22), which was published in a non-indexed database. This RCT (22) compared the efficacy of a Chinese HM with radiofrequency for the management of self-rated pain in patients diagnosed with trigeminal neuralgia (TN). The results showed that, in contrast to radiofrequency alone, the use of HM (three times daily for up to 10 days) as an adjunct to radiofrequency is more effective in reducing self-rated TN-related pain and improving the quality of life of patients with TN (22). Similar results were reported in a non-randomized clinical trial (21) that assessed the anti-nociceptive capacity of a Japanese HM (Sho-saiko-to and Keishi-ka-shakuyaku-to) in patients with TN. Although it is premature to assert that HM serves as an alternative for conventional PT, it is speculated that adjunctive use of the former alongside traditional PT helps enhance pain control in patients with TN. The authors also identified a study (31), which essentially aligned with the scope with the objectives of the present systematic review; however, this study (31) merely proposed a protocol for a future systematic review investigating the effects of HM in managing pain among patients with TN. In summary, the scarcity of high-quality clinical studies addressing the research question posed considerable challenges to conducting a clinically oriented systematic review.

### Suggestions for future research

Further well-designed and power-adjusted clinical studies (preferably RCTs) with clinically relevant dosages comparing the efficacy and safety of conventional medications (such as carbamazepine) with HM are needed to develop evidence-based guidelines for the use of HM in the management of patients with PPTTN. While the included studies (16, 17, 19, 20) demonstrated a high RoB and very low certainty of evidence on GRADE analysis, the present systematic review highlights a significant gap in the clinical evidence supporting the use of HM for the management of PPTTN. Limited scientific evidence, absence of power analyses, and methodological inconsistencies among the included studies challenged the ability to draw definitive conclusions. Therefore, the antinociceptive efficacy of HM in the management of experimentally induced PPTTN remains inconclusive. The present systematic review highlights the need for rigorous clinical investigations to determine the role of HM in the management of PPTTN.

## Conclusion

The antinociceptive efficacy of HM in the management of experimentally induced PPTTN remains inconclusive, primarily due to methodological inconsistencies within the existing literature.

## Data Availability

The original contributions presented in the study are included in the article/Supplementary Material, further inquiries can be directed to the corresponding author.
