# Supplementary material for: Efficacy of herbal medications in managing painful post-traumatic trigeminal neuropathy: a systematic review of studies in rodents
Source: Front Oral Health. 2026 Jan 21;7:1754478. doi: 10.3389/froh.2026.1754478 (PMC12868184; doi:10.3389/froh.2026.1754478)
Supplement: Supplementary file 1 [file Table1.docx]

**Appendix A:** Literature search protocol

1. Medline

| Interface: Ovid MEDLINE(R) ALL  Date of Search: 21 March 2024  Number of hits: 1,193  Comment: In Ovid, two or more words are automatically searched as phrases; i.e. no quotation marks are needed | Field labels   - exp/ = exploded MeSH term - / = non exploded MeSH term - .ti,ab,kf. = title, abstract and author keywords - adjx = within x words, regardless of order - * = truncation of word for alternate endings |
| --- | --- |
| Database(s): **Ovid MEDLINE(R) ALL**1946 to March 20, 2024 Search Strategy:   \| **#** \| **Searches** \| **Results** \| \| --- \| --- \| --- \| \| 1 \| Complementary Therapies/ \| 18377 \| \| 2 \| Drugs, Chinese Herbal/ \| 53553 \| \| 3 \| Herbal Medicine/ \| 2525 \| \| 4 \| Medicine, Kampo/ \| 775 \| \| 5 \| Medicine, Traditional/ \| 12303 \| \| 6 \| Pharmacognosy/ \| 1176 \| \| 7 \| Phytotherapy/ \| 41249 \| \| 8 \| Plant Extracts/ \| 141325 \| \| 9 \| Plant Oils/ \| 24774 \| \| 10 \| Plants, Medicinal/ \| 63078 \| \| 11 \| (alternative medicin* or alternative therap* or botanical medicin* or complementary therap* or complementary medicin* or ethno-medicin* or ethnobotan* or ethnomedicin* or folk medicin* or folk remed* or healing plant* or herb or herbaceous or herbal or herbalism or herbs or home remed* or kampo or kanpo or medicinal plant* or pharmaceutical plant* or pharmacognos* or phyto-medic* or phytomedic* or phytotherap* or plant-based medicin* or plant-based remedy or plant extract* or plant medicin* or plant oil? or primitive medicin* or saiko-keishi-to or traditional medicin* or uyakujunkisan or vegetable oil?).ti,ab,kf. \| 191252 \| \| 12 \| or/1-11 \| 400874 \| \| 13 \| Capsaicin/ or Carbamazepine/ or Chamomile/ or Capsicum/ or Corydalis/ or Garlic/ or Hypericum/ or Pueraria/ or Reishi/ \| 36822 \| \| 14 \| (allium sativum or amizepine or axsain or boletus lucidus or chili or capsaicin or capsaicine or capsicum or capsidol or capsin or capzasin or carbamazepine or carbazepin or camomil* or chamomil* or corydalis or engosaku or epitol or finlepsin or garlic? or gelcen or ganoderma lucidum or hyeonhosaek or hypericum? or katrum or kudzu? or lingzhi or matricaria or neurotol or pachyrhizus thunbergianus or pepper? or plamatine or pueraria? or puerariae? or reishi or "st. john* wort" or "st. johnswort" or saint john* wort or saint johnswort or tegretol or yan hu suo or yanhusuo or zacin or zostrix).ti,ab,kf. \| 62437 \| \| 15 \| 13 or 14 \| 67844 \| \| 16 \| abrus/ or acacia/ or acalypha/ or achillea/ or achyranthes/ or aconitum/ or acorus/ or actinidia/ or aegle/ or agave/ or ageratum/ or aloe/ or alpinia/ or ambrosia/ or ammi/ or amsinckia/ or amsonia/ or andrographis paniculata/ or anemarrhena/ or angelica/ or annona/ or anthemis/ or apocynum/ or aralia/ or arctium/ or arctostaphylos/ or argemone/ or aristolochia/ or arnica/ or artemisia/ or asarum/ or asimina/ or asparagus plant/ or aspidosperma/ or aster plant/ or atropa/ or bacopa/ or ballota/ or bassia scoparia/ or berberis/ or bidens/ or bixaceae/ or bonamia plant/ or borago/ or boswellia/ or bryonia/ or caesalpinia/ or calendula/ or calophyllum/ or calotropis/ or camellia sinensis/ or camptotheca/ or cananga/ or capsella/ or cassia/ or catha/ or catharanthus/ or caulophyllum/ or centaurium/ or cephaelis/ or chamaemelum/ or chelidonium/ or exp Chenopodium/ or chrysanthemum/ or cicuta/ or cimicifuga/ or cinchona/ or cinnamomum/ or cissus/ or cistus/ or clitoria/ or cnidium/ or coca/ or codonopsis/ or coix/ or colchicum/ or combretum/ or comfrey/ or commiphora/ or conium/ or convallaria/ or coptis chinensis/ or costus/ or crataegus/ or crocus/ or crotalaria/ or croton/ or curcuma/ or cycas/ or cyperus/ or datura metel/ or datura stramonium/ or derris/ or dioclea/ or echinacea/ or eleutherococcus/ or embelia/ or ephedra/ or epimedium/ or erigeron/ or eriodictyon/ or erysimum/ or erythrina/ or eucalyptus/ or eupatorium/ or euphorbia/ or euphorbiaceae/ or evodia/ or fallopia/ or ficus/ or forsythia/ or fucus/ or garcinia cambogia/ or garcinia kola/ or garcinia mangostana/ or gastrodia/ or glycyrrhiza/ or goniothalamus/ or gynostemma/ or hamamelis/ or harpagophytum/ or heliotropium/ or hemidesmus/ or hibiscus/ or hippophae/ or houttuynia/ or humulus/ or hydrastis/ or hydrocharitaceae/ or hymenaea/ or hyoscyamus/ or exp ilex/ or illicium/ or inula/ or juglans/ or juniperus/ or justicia/ or karaya gum/ or kava/ or lantana/ or laurus/ or lavandula/ or lawsonia plant/ or leuzea/ or ligusticum/ or lithospermum/ or lobelia/ or luffa/ or mallotus plant/ or mandragora/ or marrubium/ or marsdenia/ or matricaria/ or maytenus/ or melia azedarach/ or melilotus/ or melinis repens/ or melissa/ or mentha/ or momordica/ or monarda/ or morinda/ or moringa oleifera/ or murraya/ or myroxylon/ or myrtus/ or nelumbo/ or nepeta/ or ochrosia/ or ocimum/ or ocimum sanctum/ or oenothera/ or oplopanax/ or equisetum/ or pongamia/ or tanacetum parthenium/ or xanthium/ or origanum/ or orthosiphon/ or paeonia/ or exp panax/ or papaver/ or paris/ or parthenium hysterophorus/ or paullinia/ or peganum/ or pelargonium/ or perilla/ or peumus/ or phaseolus/ or phoradendron/ or phyllanthus/ or physostigma/ or phytolacca dodecandra/ or picrorhiza/ or pilocarpus/ or pimenta/ or pinellia/ or piper/ or piper betle/ or pistacia/ or plantago/ or plumbaginaceae/ or podophyllum/ or polyalthia/ or polygonum/ or primula/ or prosopis/ or psacalium/ or pulicaria/ or pulsatilla/ or quillaja/ or rauwolfia/ or rhamnus/ or rhodiola/ or ribes/ or ricinus communis/ or rosa/ or rubia/ or rubus/ or salacia/ or salix/ or salvia/ or sambucus/ or sanguinaria/ or sanguisorba/ or saponaria/ or sassafras/ or saussurea/ or schisandra/ or scopolia/ or scrophularia/ or scutellaria/ or senecio/ or sennosides/ or serenoa/ or silene/ or silybum marianum/ or smilax/ or solanum nigrum/ or stephania tetrandra/ or sterculia/ or stevia/ or strophanthus/ or strychnos/ or swertia/ or syzygium/ or tabebuia/ or tabernaemontana/ or tanacetum/ or taraxacum/ or taxus/ or terminalia/ or thapsia/ or tinospora/ or toxicodendron/ or tribulus/ or trichosanthes/ or trifolium/ or trigonella/ or tripterygium/ or ulex/ or uncaria/ or urtica dioica/ or vaccinium myrtillus/ or valerian/ or veratrum/ or veratrum alkaloids/ or verbascum/ or vinca/ or viscum album/ or vitex/ or vitis/ or voacanga/ or withania/ or woodfordia/ or yucca/ or zanthoxylum/ or zingiber officinale/ \| 142580 \| \| 17 \| (abrus or acacia or acalypha or achillea or achyranthes or aconite or aconitum or acorus or actaea racemosa or actinidia or aegle or aesculus or agave or ageratum or agrimonia or agrimony or alchemilla or alchornea cordifolia or alfalfa or almond or aloe or alpinia or amaryllidaceae or ambrosia or ammi or amsinckia or amsonia or anacardium or ananas or andrographis paniculata or anemarrhena or angelica or anise or annona or anthemis or apium or apocynum or apple or apricot or arachis or aralia or arctium or arctostaphylos or areca or argemone or aristolochia or armoracia or arnica or artemisia or artichoke or asarum or asimina or asparagus or aspidosperma or aster plant or astragalus or atropa or avocado or bacopa or ballota or barley or basil or bassia scoparia or bayberry or beet or belladonna or berberis or beta vulgaris or betel palm or bidens or bilberry or bistorta officinalis or bixa orellana or bixaceae or black cohosh or black currant or blackberry or blueberry or bonamia angiosperm or bonamia plant or borago or boswellia or brassica or broccoli or bryonia or buckthorn or butterbur or cabbage or cacao or caesalpinia or calendula or calophyllum or calotropis or camellia sinensis or camphor or camptotheca or cananga or cannabis or capsella or capsicum or carica or carrot or cascara or cashew or cassia or cat* claw or catha or catharanthus or catnip or caulophyllum or cayenne pepper or celery or centaurium or centella asiatica or cephaelis or chamaemelum or chamomile or chaparral or chelidonium or chenopodium or chive or chlorophytum borivilianum or chrysanthemum or cicuta or cimicifuga or cinchona or cinnamomum or cinnamon or cissus or cistus or citrus or clitoria or cnidium or coca or coconut or cocos or codonopsis or coffee or coix or colchicum or combretum or comfrey or commiphora or condurango or conium or convallaria or coptis chinensis or corn or costus or cranberry or crataegus or crocus or crotalaria or croton or cucurbita or curcuma or cycas or cymbopogon or cynara scolymus or cyperus or dandelion or datura metel or daucus carota or derris or devil's claw or dioclea or dioscorea or dong quai or ecballium elaterium or echinacea or elderberry or eleutherococcus or embelia or ephedra or epimedium or equisetum or eremophila angiosperm or erigeron or eriodictyon or erysimum or erythrina or eucalyptus or eupatorium or euphorbia or euphorbiaceae or euterpe or evening primrose or evodia or fallopia or fennel or fenugreek or feverfew or ficus or fig or flax or flaxseed or foeniculum or forsythia or fragaria or frangula or fucus or garcinia cambogia or garcinia kola or garcinia mangostana or gastrodia or ginger or ginkgo biloba or ginseng or glycine max or glycyrrhiza or goldenseal or goniothalamus or grape or grapefruit or green tea or guarana or guava or gynostemma or hamamelis or harpagophytum or hawthorn or heliotropium or hemidesmus or hibiscus or hippophae or hops or hordeum or horse chestnut or horseradish or houttuynia or humulus or hydrastis or hydrocharitaceae or hymenaea or hyoscyamus or ilex or illicium or inula or ivy or juglans or juniper or juniperus or justicia or karaya gum or kava or kiwi or kochia scoparia or lady* mantle or lagenaria siceraria or lantana or laurus or lavandula or lavender or lawsonia inermis or lawsonia plant or lemon or leuzea or licorice or ligusticum or "lily of the valley" or lime or lithospermum or lobelia or luffa or macrotyloma uniflorum or mallotus philippinensis or mallotus plant or malus or mandragora or marigold or maritime squill or marrubium or marsdenia or matricaria or maytenus or medicago sativa or melaleuca or melia azedarach or melilotus or melinis repens or melissa or mentha or milk thistle or mint or mistletoe or momordica or monarda or morinda or moringa oleifera or mugwort or murraya or mustard or myrica or myristica or myroxylon or myrtle or myrtus or nelumbo or nepeta or nerium or noni or nutmeg or oat or ochrosia or ocimum or oenothera or olea or oleander or olive or onions or oplopanax or oregano or origanum or oroxylum or orthosiphon or paeonia or panax or papaver or papaya or paris or parsley or parsnip or parthenium hysterophorus or passiflora or passionflower or pastinaca or paullinia or pausinystalia or peach or peanut or pear or peganum or pelargonium or pennyroyal or perilla or persea or petasites or petroselinum or peumus or phaseolus or phoradendron or phyllanthus or physostigma or phytolacca dodecandra or picrorhiza or pilocarpus or pimenta or pineapple or pinellia or piper or pistacia or plantago or plumbaginaceae or plumbago zeylanica or podophyllum or poison ivy or polyalthia or polygonum or pomegranate or pongamia or potato or primula or prosopis or prunus or psacalium or psidium or psyllium or pulicaria or pulsatilla or pumpkin or pygeum or pyrus or quillaja or radish or ragwort or raphanus or raspberry or rauvolfia or rauwolfia or red clover or rhamnus or rheum or rhodiola or rhubarb or ribes or ricinus communis or rosa or rose or rosemary or rubia or rubus or sabal or sage or salacia or salix or salvia or sambucus or sanguinaria or sanguisorba or saponaria or sarsaparilla or sassafras or saussurea or saw palmetto or schinus or schisandra or scopolia or scrophularia or scutellaria or senecio or senegalia catechu or senegalia modesta or senegalia senegal or senna or sennosides or serenoa or silene or silybum marianum or slippery elm or smallanthus sonchifolius or smilax or solanum lycopersicum or solanum nigrum or solanum tuberosum or sour cherry or soybeans or spinach or spinacia oleracea or squash or stephania tetrandra or sterculia or stevia or stinging nettle or strawberry or strophanthus or strychnos or swertia or syzygium or tabebuia or tabernaemontana or talus or tanacetum or taraxacum or taxus or tea tree or terminalia or thapsia or thyme or thymus plant or tinospora or tobacco or tomatoes or toxicodendron or tribulus or trichosanthes or tridax procumbens or trifolium or trigonella or tripterygium or turmeric or ulex or ulmus or uncaria or urtica dioica or vaccinium macrocarpon or vaccinium myrtillus or vachellia farnesiana or vachellia nilotica or vachellia tortilis or valerian or valeriana or veratrum or verbascum or vinca or viscum album or vitex or vitis or voacanga or wild cherry or wild yam or willow or witch hazel or withania or woodfordia or wrightia tinctoria or wrightia tomentosa or xanthium or xanthorrhiza or yarrow or yew or yohimbe or yucca or zanthoxylum or zataria multiflora or zea mays or zingiber officinale).ti,ab,kf. \| 908387 \| \| 18 \| 16 or 17 \| 934797 \| \| 19 \| 12 or 15 or 18 \| 1232598 \| \| 20 \| Trigeminal Neuralgia/ \| 7821 \| \| 21 \| Trigeminal Nerve Diseases/ \| 1065 \| \| 22 \| (drigeminal neuropath* or epileptiform neuralg* or fothergill disease* or prosopalgia or tic doloureux or tic douloureux or trifacial neuralg* or trigemin* neuralg* or trigemin* nerve disease* or trigemin* nerve neuralgia or trigemin* nerve disorder* or trigemin* neuropathic pain).ti,ab,kf. \| 8421 \| \| 23 \| ((trigemin* nerve or mandibul* nerve or mandibul* neuropathic or maxilla* nerve or chronic neuralgia) and pain).ti,ab,kf. \| 2641 \| \| 24 \| or/20-23 \| 12445 \| \| 25 \| 19 and 24 \| 1193 \| | |

2. Embase

| Interface: embase.com  Date of Search: 21 March 2024  Number of hits: 3,243  Comment: Emtree is the controlled vocabulary in Embase | Field labels   - /exp = exploded Emtree term - /de = non exploded Emtree term - ti,ab,kw = title, abstract and author keywords - NEAR/x = within x words, regardless of order - * = truncation of word for alternate endings |
| --- | --- |
| \| **No.** \| **Query** \| **Results** \| \| --- \| --- \| --- \| \| #1 \| 'alternative medicine'/de \| 52772 \| \| #2 \| 'herbaceous agent'/de \| 61997 \| \| #3 \| 'herbal medicine'/de \| 30145 \| \| #4 \| 'kampo medicine'/de \| 597 \| \| #5 \| 'traditional medicine'/de \| 35307 \| \| #6 \| 'pharmacognosy'/de \| 4435 \| \| #7 \| 'phytotherapy'/de \| 19246 \| \| #8 \| 'plant extract'/de \| 191275 \| \| #9 \| 'vegetable oil'/de \| 20521 \| \| #10 \| 'medicinal plant'/de \| 100447 \| \| #11 \| 'alternative medicin*':ti,ab,kw OR 'alternative therap*':ti,ab,kw OR 'botanical medicin*':ti,ab,kw OR 'ethno medicin*':ti,ab,kw OR 'complementary therap*':ti,ab,kw OR 'complementary medicin*':ti,ab,kw OR ethnobotan*:ti,ab,kw OR ethnomedicin*:ti,ab,kw OR 'folk medicin*':ti,ab,kw OR 'folk remed*':ti,ab,kw OR 'healing plant*':ti,ab,kw OR herb:ti,ab,kw OR herbaceous:ti,ab,kw OR herbal:ti,ab,kw OR herbalism:ti,ab,kw OR herbs:ti,ab,kw OR 'home remed*':ti,ab,kw OR kampo:ti,ab,kw OR kanpo:ti,ab,kw OR 'medicinal plant*':ti,ab,kw OR 'pharmaceutical plant*':ti,ab,kw OR pharmacognos*:ti,ab,kw OR 'phyto medic*':ti,ab,kw OR phytomedic*:ti,ab,kw OR phytotherap*:ti,ab,kw OR 'plant based medicin*':ti,ab,kw OR 'plant based remedy':ti,ab,kw OR 'plant extract*':ti,ab,kw OR 'plant medicin*':ti,ab,kw OR plant-oil$:ti,ab,kw OR 'primitive medicin*':ti,ab,kw OR 'saiko keishi to':ti,ab,kw OR 'traditional medicin*':ti,ab,kw OR uyakujunkisan:ti,ab,kw OR 'vegetable oil$':ti,ab,kw \| 281420 \| \| #12 \| #1 OR #2 OR #3 OR #4 OR #5 OR #6 OR #7 OR #8 OR #9 OR #10 OR #11 \| 532411 \| \| #13 \| 'capsaicin'/de OR 'carbamazepine'/de OR 'ganoderma lucidum'/exp OR 'pepper'/exp OR 'pueraria'/exp \| 108929 \| \| #14 \| 'allium sativum':ti,ab,kw OR amizepine:ti,ab,kw OR axsain:ti,ab,kw OR 'boletus lucidus':ti,ab,kw OR chili:ti,ab,kw OR capsaicin:ti,ab,kw OR capsaicine:ti,ab,kw OR capsicum:ti,ab,kw OR capsidol:ti,ab,kw OR capsin:ti,ab,kw OR capzasin:ti,ab,kw OR carbamazepine:ti,ab,kw OR carbazepin:ti,ab,kw OR camomil*:ti,ab,kw OR chamomil*:ti,ab,kw OR corydalis:ti,ab,kw OR engosaku:ti,ab,kw OR epitol:ti,ab,kw OR finlepsin:ti,ab,kw OR garlic$:ti,ab,kw OR gelcen:ti,ab,kw OR 'ganoderma lucidum':ti,ab,kw OR hyeonhosaek:ti,ab,kw OR hypericum$:ti,ab,kw OR katrum:ti,ab,kw OR kudzu$:ti,ab,kw OR lingzhi:ti,ab,kw OR matricaria:ti,ab,kw OR neurotol:ti,ab,kw OR 'pachyrhizus thunbergianus':ti,ab,kw OR pepper$:ti,ab,kw OR plamatine:ti,ab,kw OR pueraria$:ti,ab,kw OR puerariae$:ti,ab,kw OR reishi:ti,ab,kw OR 'st. johnswort':ti,ab,kw OR 'saint johnswort':ti,ab,kw OR 'st. john* wort':ti,ab,kw OR 'saint john* wort':ti,ab,kw OR tegretol:ti,ab,kw OR 'yan hu suo':ti,ab,kw OR yanhusuo:ti,ab,kw OR zacin:ti,ab,kw OR zostrix:ti,ab,kw \| 79397 \| \| #15 \| #13 OR #14 \| 139089 \| \| #16 \| 'medicinal plant'/exp \| 322220 \| \| #17 \| abrus:ti,ab,kw OR acacia:ti,ab,kw OR acalypha:ti,ab,kw OR achillea:ti,ab,kw OR achyranthes:ti,ab,kw OR aconite:ti,ab,kw OR aconitum:ti,ab,kw OR acorus:ti,ab,kw OR 'actaea racemosa':ti,ab,kw OR actinidia:ti,ab,kw OR aegle:ti,ab,kw OR aesculus:ti,ab,kw OR agave:ti,ab,kw OR ageratum:ti,ab,kw OR agrimonia:ti,ab,kw OR agrimony:ti,ab,kw OR alchemilla:ti,ab,kw OR 'alchornea cordifolia':ti,ab,kw OR alfalfa:ti,ab,kw OR almond:ti,ab,kw OR aloe:ti,ab,kw OR alpinia:ti,ab,kw OR amaryllidaceae:ti,ab,kw OR ambrosia:ti,ab,kw OR ammi:ti,ab,kw OR amsinckia:ti,ab,kw OR amsonia:ti,ab,kw OR anacardium:ti,ab,kw OR ananas:ti,ab,kw OR 'andrographis paniculata':ti,ab,kw OR anemarrhena:ti,ab,kw OR angelica:ti,ab,kw OR anise:ti,ab,kw OR annona:ti,ab,kw OR anthemis:ti,ab,kw OR apium:ti,ab,kw OR apocynum:ti,ab,kw OR apple:ti,ab,kw OR apricot:ti,ab,kw OR arachis:ti,ab,kw OR aralia:ti,ab,kw OR arctium:ti,ab,kw OR arctostaphylos:ti,ab,kw OR areca:ti,ab,kw OR argemone:ti,ab,kw OR aristolochia:ti,ab,kw OR armoracia:ti,ab,kw OR arnica:ti,ab,kw OR artemisia:ti,ab,kw OR artichoke:ti,ab,kw OR asarum:ti,ab,kw OR asimina:ti,ab,kw OR asparagus:ti,ab,kw OR aspidosperma:ti,ab,kw OR 'aster plant':ti,ab,kw OR astragalus:ti,ab,kw OR atropa:ti,ab,kw OR avocado:ti,ab,kw OR bacopa:ti,ab,kw OR ballota:ti,ab,kw OR barley:ti,ab,kw OR basil:ti,ab,kw OR 'bassia scoparia':ti,ab,kw OR bayberry:ti,ab,kw OR beet:ti,ab,kw OR belladonna:ti,ab,kw OR berberis:ti,ab,kw OR 'beta vulgaris':ti,ab,kw OR 'betel palm':ti,ab,kw OR bidens:ti,ab,kw OR bilberry:ti,ab,kw OR 'bistorta officinalis':ti,ab,kw OR 'bixa orellana':ti,ab,kw OR bixaceae:ti,ab,kw OR 'black cohosh':ti,ab,kw OR 'black currant':ti,ab,kw OR blackberry:ti,ab,kw OR blueberry:ti,ab,kw OR 'bonamia angiosperm':ti,ab,kw OR 'bonamia plant':ti,ab,kw OR borago:ti,ab,kw OR boswellia:ti,ab,kw OR brassica:ti,ab,kw OR broccoli:ti,ab,kw OR bryonia:ti,ab,kw OR buckthorn:ti,ab,kw OR butterbur:ti,ab,kw OR cabbage:ti,ab,kw OR cacao:ti,ab,kw OR caesalpinia:ti,ab,kw OR calendula:ti,ab,kw OR calophyllum:ti,ab,kw OR calotropis:ti,ab,kw OR 'camellia sinensis':ti,ab,kw OR camphor:ti,ab,kw OR camptotheca:ti,ab,kw OR cananga:ti,ab,kw OR cannabis:ti,ab,kw OR capsella:ti,ab,kw OR capsicum:ti,ab,kw OR carica:ti,ab,kw OR carrot:ti,ab,kw OR cascara:ti,ab,kw OR cashew:ti,ab,kw OR cassia:ti,ab,kw OR 'cat* claw':ti,ab,kw OR catha:ti,ab,kw OR catharanthus:ti,ab,kw OR catnip:ti,ab,kw OR caulophyllum:ti,ab,kw OR 'cayenne pepper':ti,ab,kw OR celery:ti,ab,kw OR centaurium:ti,ab,kw OR 'centella asiatica':ti,ab,kw OR cephaelis:ti,ab,kw OR chamaemelum:ti,ab,kw OR chamomile:ti,ab,kw OR chaparral:ti,ab,kw OR chelidonium:ti,ab,kw OR chenopodium:ti,ab,kw OR chive:ti,ab,kw OR 'chlorophytum borivilianum':ti,ab,kw OR chrysanthemum:ti,ab,kw OR cicuta:ti,ab,kw OR cimicifuga:ti,ab,kw OR cinchona:ti,ab,kw OR cinnamomum:ti,ab,kw OR cinnamon:ti,ab,kw OR cissus:ti,ab,kw OR cistus:ti,ab,kw OR citrus:ti,ab,kw OR clitoria:ti,ab,kw OR cnidium:ti,ab,kw OR coca:ti,ab,kw OR coconut:ti,ab,kw OR cocos:ti,ab,kw OR codonopsis:ti,ab,kw OR coffee:ti,ab,kw OR coix:ti,ab,kw OR colchicum:ti,ab,kw OR combretum:ti,ab,kw OR comfrey:ti,ab,kw OR commiphora:ti,ab,kw OR condurango:ti,ab,kw OR conium:ti,ab,kw OR convallaria:ti,ab,kw OR 'coptis chinensis':ti,ab,kw OR corn:ti,ab,kw OR costus:ti,ab,kw OR cranberry:ti,ab,kw OR crataegus:ti,ab,kw OR crocus:ti,ab,kw OR crotalaria:ti,ab,kw OR croton:ti,ab,kw OR cucurbita:ti,ab,kw OR curcuma:ti,ab,kw OR cycas:ti,ab,kw OR cymbopogon:ti,ab,kw OR 'cynara scolymus':ti,ab,kw OR cyperus:ti,ab,kw OR dandelion:ti,ab,kw OR 'datura metel':ti,ab,kw OR 'daucus carota':ti,ab,kw OR derris:ti,ab,kw OR 'devil* claw':ti,ab,kw OR dioclea:ti,ab,kw OR dioscorea:ti,ab,kw OR 'dong quai':ti,ab,kw OR 'ecballium elaterium':ti,ab,kw OR echinacea:ti,ab,kw OR elderberry:ti,ab,kw OR eleutherococcus:ti,ab,kw OR embelia:ti,ab,kw OR ephedra:ti,ab,kw OR epimedium:ti,ab,kw OR equisetum:ti,ab,kw OR 'eremophila angiosperm':ti,ab,kw OR erigeron:ti,ab,kw OR eriodictyon:ti,ab,kw OR erysimum:ti,ab,kw OR erythrina:ti,ab,kw OR eucalyptus:ti,ab,kw OR eupatorium:ti,ab,kw OR euphorbia:ti,ab,kw OR euphorbiaceae:ti,ab,kw OR euterpe:ti,ab,kw OR 'evening primrose':ti,ab,kw OR evodia:ti,ab,kw OR fallopia:ti,ab,kw OR fennel:ti,ab,kw OR fenugreek:ti,ab,kw OR feverfew:ti,ab,kw OR ficus:ti,ab,kw OR fig:ti,ab,kw OR flax:ti,ab,kw OR flaxseed:ti,ab,kw OR foeniculum:ti,ab,kw OR forsythia:ti,ab,kw OR fragaria:ti,ab,kw OR frangula:ti,ab,kw OR fucus:ti,ab,kw OR 'garcinia cambogia':ti,ab,kw OR 'garcinia kola':ti,ab,kw OR 'garcinia mangostana':ti,ab,kw OR gastrodia:ti,ab,kw OR ginger:ti,ab,kw OR 'ginkgo biloba':ti,ab,kw OR ginseng:ti,ab,kw OR 'glycine max':ti,ab,kw OR glycyrrhiza:ti,ab,kw OR goldenseal:ti,ab,kw OR goniothalamus:ti,ab,kw OR grape:ti,ab,kw OR grapefruit:ti,ab,kw OR 'green tea':ti,ab,kw OR guarana:ti,ab,kw OR guava:ti,ab,kw OR gynostemma:ti,ab,kw OR hamamelis:ti,ab,kw OR harpagophytum:ti,ab,kw OR hawthorn:ti,ab,kw OR heliotropium:ti,ab,kw OR hemidesmus:ti,ab,kw OR hibiscus:ti,ab,kw OR hippophae:ti,ab,kw OR hops:ti,ab,kw OR hordeum:ti,ab,kw OR 'horse chestnut':ti,ab,kw OR horseradish:ti,ab,kw OR houttuynia:ti,ab,kw OR humulus:ti,ab,kw OR hydrastis:ti,ab,kw OR hydrocharitaceae:ti,ab,kw OR hymenaea:ti,ab,kw OR hyoscyamus:ti,ab,kw OR ilex:ti,ab,kw OR illicium:ti,ab,kw OR inula:ti,ab,kw OR ivy:ti,ab,kw OR juglans:ti,ab,kw OR juniper:ti,ab,kw OR juniperus:ti,ab,kw OR justicia:ti,ab,kw OR 'karaya gum':ti,ab,kw OR kava:ti,ab,kw OR kiwi:ti,ab,kw OR 'kochia scoparia':ti,ab,kw OR 'lady* mantle':ti,ab,kw OR 'lagenaria siceraria':ti,ab,kw OR lantana:ti,ab,kw OR laurus:ti,ab,kw OR lavandula:ti,ab,kw OR lavender:ti,ab,kw OR 'lawsonia inermis':ti,ab,kw OR 'lawsonia plant':ti,ab,kw OR lemon:ti,ab,kw OR leuzea:ti,ab,kw OR licorice:ti,ab,kw OR ligusticum:ti,ab,kw OR 'lily of the valley':ti,ab,kw OR lime:ti,ab,kw OR lithospermum:ti,ab,kw OR lobelia:ti,ab,kw OR luffa:ti,ab,kw OR 'macrotyloma uniflorum':ti,ab,kw OR 'mallotus philippinensis':ti,ab,kw OR 'mallotus plant':ti,ab,kw OR malus:ti,ab,kw OR mandragora:ti,ab,kw OR marigold:ti,ab,kw OR 'maritime squill':ti,ab,kw OR marrubium:ti,ab,kw OR marsdenia:ti,ab,kw OR matricaria:ti,ab,kw OR maytenus:ti,ab,kw OR 'medicago sativa':ti,ab,kw OR melaleuca:ti,ab,kw OR 'melia azedarach':ti,ab,kw OR melilotus:ti,ab,kw OR 'melinis repens':ti,ab,kw OR melissa:ti,ab,kw OR mentha:ti,ab,kw OR 'milk thistle':ti,ab,kw OR mint:ti,ab,kw OR mistletoe:ti,ab,kw OR momordica:ti,ab,kw OR monarda:ti,ab,kw OR morinda:ti,ab,kw OR 'moringa oleifera':ti,ab,kw OR mugwort:ti,ab,kw OR murraya:ti,ab,kw OR mustard:ti,ab,kw OR myrica:ti,ab,kw OR myristica:ti,ab,kw OR myroxylon:ti,ab,kw OR myrtle:ti,ab,kw OR myrtus:ti,ab,kw OR nelumbo:ti,ab,kw OR nepeta:ti,ab,kw OR nerium:ti,ab,kw OR noni:ti,ab,kw OR nutmeg:ti,ab,kw OR oat:ti,ab,kw OR ochrosia:ti,ab,kw OR ocimum:ti,ab,kw OR oenothera:ti,ab,kw OR olea:ti,ab,kw OR oleander:ti,ab,kw OR olive:ti,ab,kw OR onions:ti,ab,kw OR oplopanax:ti,ab,kw OR oregano:ti,ab,kw OR origanum:ti,ab,kw OR oroxylum:ti,ab,kw OR orthosiphon:ti,ab,kw OR paeonia:ti,ab,kw OR panax:ti,ab,kw OR papaver:ti,ab,kw OR papaya:ti,ab,kw OR paris:ti,ab,kw OR parsley:ti,ab,kw OR parsnip:ti,ab,kw OR 'parthenium hysterophorus':ti,ab,kw OR passiflora:ti,ab,kw OR passionflower:ti,ab,kw OR pastinaca:ti,ab,kw OR paullinia:ti,ab,kw OR pausinystalia:ti,ab,kw OR peach:ti,ab,kw OR peanut:ti,ab,kw OR pear:ti,ab,kw OR peganum:ti,ab,kw OR pelargonium:ti,ab,kw OR pennyroyal:ti,ab,kw OR perilla:ti,ab,kw OR persea:ti,ab,kw OR petasites:ti,ab,kw OR petroselinum:ti,ab,kw OR peumus:ti,ab,kw OR phaseolus:ti,ab,kw OR phoradendron:ti,ab,kw OR phyllanthus:ti,ab,kw OR physostigma:ti,ab,kw OR 'phytolacca dodecandra':ti,ab,kw OR picrorhiza:ti,ab,kw OR pilocarpus:ti,ab,kw OR pimenta:ti,ab,kw OR pineapple:ti,ab,kw OR pinellia:ti,ab,kw OR piper:ti,ab,kw OR pistacia:ti,ab,kw OR plantago:ti,ab,kw OR plumbaginaceae:ti,ab,kw OR 'plumbago zeylanica':ti,ab,kw OR podophyllum:ti,ab,kw OR 'poison ivy':ti,ab,kw OR polyalthia:ti,ab,kw OR polygonum:ti,ab,kw OR pomegranate:ti,ab,kw OR pongamia:ti,ab,kw OR potato:ti,ab,kw OR primula:ti,ab,kw OR prosopis:ti,ab,kw OR prunus:ti,ab,kw OR psacalium:ti,ab,kw OR psidium:ti,ab,kw OR psyllium:ti,ab,kw OR pulicaria:ti,ab,kw OR pulsatilla:ti,ab,kw OR pumpkin:ti,ab,kw OR pygeum:ti,ab,kw OR pyrus:ti,ab,kw OR quillaja:ti,ab,kw OR radish:ti,ab,kw OR ragwort:ti,ab,kw OR raphanus:ti,ab,kw OR raspberry:ti,ab,kw OR rauvolfia:ti,ab,kw OR rauwolfia:ti,ab,kw OR 'red clover':ti,ab,kw OR rhamnus:ti,ab,kw OR rheum:ti,ab,kw OR rhodiola:ti,ab,kw OR rhubarb:ti,ab,kw OR ribes:ti,ab,kw OR 'ricinus communis':ti,ab,kw OR rosa:ti,ab,kw OR rose:ti,ab,kw OR rosemary:ti,ab,kw OR rubia:ti,ab,kw OR rubus:ti,ab,kw OR sabal:ti,ab,kw OR sage:ti,ab,kw OR salacia:ti,ab,kw OR salix:ti,ab,kw OR salvia:ti,ab,kw OR sambucus:ti,ab,kw OR sanguinaria:ti,ab,kw OR sanguisorba:ti,ab,kw OR saponaria:ti,ab,kw OR sarsaparilla:ti,ab,kw OR sassafras:ti,ab,kw OR saussurea:ti,ab,kw OR 'saw palmetto':ti,ab,kw OR schinus:ti,ab,kw OR schisandra:ti,ab,kw OR scopolia:ti,ab,kw OR scrophularia:ti,ab,kw OR scutellaria:ti,ab,kw OR senecio:ti,ab,kw OR 'senegalia catechu':ti,ab,kw OR 'senegalia modesta':ti,ab,kw OR 'senegalia senegal':ti,ab,kw OR senna:ti,ab,kw OR sennosides:ti,ab,kw OR serenoa:ti,ab,kw OR silene:ti,ab,kw OR 'silybum marianum':ti,ab,kw OR 'slippery elm':ti,ab,kw OR 'smallanthus sonchifolius':ti,ab,kw OR smilax:ti,ab,kw OR 'solanum lycopersicum':ti,ab,kw OR 'solanum nigrum':ti,ab,kw OR 'solanum tuberosum':ti,ab,kw OR 'sour cherry':ti,ab,kw OR soybeans:ti,ab,kw OR spinach:ti,ab,kw OR 'spinacia oleracea':ti,ab,kw OR squash:ti,ab,kw OR 'stephania tetrandra':ti,ab,kw OR sterculia:ti,ab,kw OR stevia:ti,ab,kw OR 'stinging nettle':ti,ab,kw OR strawberry:ti,ab,kw OR strophanthus:ti,ab,kw OR strychnos:ti,ab,kw OR swertia:ti,ab,kw OR syzygium:ti,ab,kw OR tabebuia:ti,ab,kw OR tabernaemontana:ti,ab,kw OR talus:ti,ab,kw OR tanacetum:ti,ab,kw OR taraxacum:ti,ab,kw OR taxus:ti,ab,kw OR 'tea tree':ti,ab,kw OR terminalia:ti,ab,kw OR thapsia:ti,ab,kw OR thyme:ti,ab,kw OR 'thymus plant':ti,ab,kw OR tinospora:ti,ab,kw OR tobacco:ti,ab,kw OR tomatoes:ti,ab,kw OR toxicodendron:ti,ab,kw OR tribulus:ti,ab,kw OR trichosanthes:ti,ab,kw OR 'tridax procumbens':ti,ab,kw OR trifolium:ti,ab,kw OR trigonella:ti,ab,kw OR tripterygium:ti,ab,kw OR turmeric:ti,ab,kw OR ulex:ti,ab,kw OR ulmus:ti,ab,kw OR uncaria:ti,ab,kw OR 'urtica dioica':ti,ab,kw OR 'vaccinium macrocarpon':ti,ab,kw OR 'vaccinium myrtillus':ti,ab,kw OR 'vachellia farnesiana':ti,ab,kw OR 'vachellia nilotica':ti,ab,kw OR 'vachellia tortilis':ti,ab,kw OR valerian:ti,ab,kw OR valeriana:ti,ab,kw OR veratrum:ti,ab,kw OR verbascum:ti,ab,kw OR vinca:ti,ab,kw OR 'viscum album':ti,ab,kw OR vitex:ti,ab,kw OR vitis:ti,ab,kw OR voacanga:ti,ab,kw OR 'wild cherry':ti,ab,kw OR 'wild yam':ti,ab,kw OR willow:ti,ab,kw OR 'witch hazel':ti,ab,kw OR withania:ti,ab,kw OR woodfordia:ti,ab,kw OR 'wrightia tinctoria':ti,ab,kw OR 'wrightia tomentosa':ti,ab,kw OR xanthium:ti,ab,kw OR xanthorrhiza:ti,ab,kw OR yarrow:ti,ab,kw OR yew:ti,ab,kw OR yohimbe:ti,ab,kw OR yucca:ti,ab,kw OR zanthoxylum:ti,ab,kw OR 'zataria multiflora':ti,ab,kw OR 'zea mays':ti,ab,kw OR 'zingiber officinale':ti,ab,kw \| 1189861 \| \| #18 \| #16 OR #17 \| 1312935 \| \| #19 \| #12 OR #15 OR #18 \| 1694475 \| \| #20 \| 'trigeminus neuralgia'/exp \| 14453 \| \| #21 \| 'trigeminal nerve disease'/de \| 1304 \| \| #22 \| 'drigeminal neuropath*':ti,ab,kw OR 'epileptiform neuralg*':ti,ab,kw OR 'fothergill disease*':ti,ab,kw OR prosopalgia:ti,ab,kw OR 'tic doloureux':ti,ab,kw OR 'tic douloureux':ti,ab,kw OR 'trifacial neuralg*':ti,ab,kw OR 'trigemin* neuralg*':ti,ab,kw OR 'trigemin* nerve disease*':ti,ab,kw OR 'trigemin* nerve neuralgia':ti,ab,kw OR 'trigemin* nerve disorder*':ti,ab,kw OR 'trigemin* neuropathic pain':ti,ab,kw \| 10812 \| \| #23 \| ('trigemin* nerve':ti,ab,kw OR 'mandibul* nerve':ti,ab,kw OR 'mandibul* neuropathic':ti,ab,kw OR 'maxilla* nerve':ti,ab,kw OR 'chronic neuralgia':ti,ab,kw) AND pain:ti,ab,kw \| 3826 \| \| #24 \| #20 OR #21 OR #22 OR #23 \| 18667 \| \| #25 \| #19 AND #24 \| 3560 \| \| #26 \| #25 AND 'Conference Abstract'/it \| 317 \| \| #27 \| #25 NOT #26 \| 3243 \| | |

3. Cochrane Library

| Interface: Wiley  Date of Search: 21 March 2024  Number of hits: 149 | Field labels   - ti,ab,kw = title, abstract and author keywords - NEAR/x = within x words, regardless of order - * = truncation of word for alternate endings |
| --- | --- |
| \| ID \| Search \| Hits \| \| --- \| --- \| --- \| \| #1 \| [mh ^"Complementary Therapies"] \| 630 \| \| #2 \| [mh ^"Drugs, Chinese Herbal"] \| 4594 \| \| #3 \| [mh ^"Herbal Medicine"] \| 100 \| \| #4 \| [mh ^"Medicine, Kampo"] \| 67 \| \| #5 \| [mh ^"Medicine, Traditional"] \| 133 \| \| #6 \| [mh ^"Pharmacognosy"] \| 0 \| \| #7 \| [mh ^"Phytotherapy"] \| 4647 \| \| #8 \| [mh ^"Plant Extracts"] \| 5708 \| \| #9 \| [mh ^"Plant Oils"] \| 1619 \| \| #10 \| [mh ^"Plants, Medicinal"] \| 1149 \| \| #11 \| (("alternative" NEXT medicin*) OR ("alternative" NEXT therap*) OR ("botanical" NEXT medicin*) OR ("complementary" NEXT therap*) OR ("complementary" NEXT medicin*) OR ethno-medicin* OR ethnobotan* OR ethnomedicin* OR ("folk" NEXT medicin*) OR ("folk" NEXT remed*) OR ("healing" NEXT plant*) OR herb OR herbaceous OR herbal OR herbalism OR herbs OR ("home" NEXT remed*) OR kampo OR kanpo OR ("medicinal" NEXT plant*) OR ("pharmaceutical" NEXT plant*) OR pharmacognos* OR phyto-medic* OR phytomedic* OR phytotherap* OR ("plant-based" NEXT medicin*) OR "plant-based remedy" OR ("plant" NEXT extract*) OR ("plant" NEXT medicin*) OR ("plant" NEXT oil?) OR ("primitive" NEXT medicin*) OR saiko-keishi-to OR ("traditional" NEXT medicin*) OR uyakujunkisan OR ("vegetable" NEXT oil?) ):ti,ab,kw \| 29917 \| \| #12 \| #1 OR #2 OR #3 OR #4 OR #5 OR #6 OR #7 OR #8 OR #9 OR #10 OR #11 \| 30284 \| \| #13 \| [mh ^Capsaicin] OR [mh ^Carbamazepine] OR [mh ^Chamomile] OR [mh ^Capsicum] OR [mh ^Corydalis] OR [mh ^Garlic] OR [mh ^Hypericum] OR [mh ^Pueraria] OR [mh ^Reishi] \| 2384 \| \| #14 \| ("allium sativum" OR amizepine OR axsain OR "boletus lucidus" OR chili OR capsaicin OR capsaicine OR capsicum OR capsidol OR capsin OR capzasin OR carbamazepine OR carbazepin OR camomil* OR chamomil* OR corydalis OR engosaku OR epitol OR finlepsin OR garlic? OR gelcen OR "ganoderma lucidum" OR hyeonhosaek OR hypericum? OR katrum OR kudzu? OR lingzhi OR matricaria OR neurotol OR "pachyrhizus thunbergianus" OR pepper? OR plamatine OR pueraria? OR puerariae? OR reishi OR ("st." NEXT john* NEXT "wort") OR "st. johnswort" OR ("saint" NEXT john* NEXT "wort") OR "saint johnswort" OR tegretol OR "yan hu suo" OR yanhusuo OR zacin OR zostrix ):ti,ab,kw \| 7478 \| \| #15 \| #13 OR #14 \| 7478 \| \| #16 \| [mh ^abrus] OR [mh ^acacia] OR [mh ^acalypha] OR [mh ^achillea] OR [mh ^achyranthes] OR [mh ^aconitum] OR [mh ^acorus] OR [mh ^actinidia] OR [mh ^aegle] OR [mh ^agave] OR [mh ^ageratum] OR [mh ^aloe] OR [mh ^alpinia] OR [mh ^ambrosia] OR [mh ^ammi] OR [mh ^amsinckia] OR [mh ^amsonia] OR [mh ^"andrographis paniculata"] OR [mh ^anemarrhena] OR [mh ^angelica] OR [mh ^annona] OR [mh ^anthemis] OR [mh ^apocynum] OR [mh ^aralia] OR [mh ^arctium] OR [mh ^arctostaphylos] OR [mh ^argemone] OR [mh ^aristolochia] OR [mh ^arnica] OR [mh ^artemisia] OR [mh ^asarum] OR [mh ^asimina] OR [mh ^"asparagus plant"] OR [mh ^aspidosperma] OR [mh ^"aster plant"] OR [mh ^atropa] OR [mh ^bacopa] OR [mh ^ballota] OR [mh ^"bassia scoparia"] OR [mh ^berberis] OR [mh ^bidens] OR [mh ^bixaceae] OR [mh ^"bonamia plant"] OR [mh ^borago] OR [mh ^boswellia] OR [mh ^bryonia] OR [mh ^caesalpinia] OR [mh ^calendula] OR [mh ^calophyllum] OR [mh ^calotropis] OR [mh ^"camellia sinensis"] OR [mh ^camptotheca] OR [mh ^cananga] OR [mh ^capsella] OR [mh ^cassia] OR [mh ^catha] OR [mh ^catharanthus] OR [mh ^caulophyllum] OR [mh ^centaurium] OR [mh ^cephaelis] OR [mh ^chamaemelum] OR [mh ^chelidonium] OR [mh Chenopodium] OR [mh ^chrysanthemum] OR [mh ^cicuta] OR [mh ^cimicifuga] OR [mh ^cinchona] OR [mh ^cinnamomum] OR [mh ^cissus] OR [mh ^cistus] OR [mh ^clitoria] OR [mh ^cnidium] OR [mh ^coca] OR [mh ^codonopsis] OR [mh ^coix] OR [mh ^colchicum] OR [mh ^combretum] OR [mh ^comfrey] OR [mh ^commiphora] OR [mh ^conium] OR [mh ^convallaria] OR [mh ^"coptis chinensis"] OR [mh ^costus] OR [mh ^crataegus] OR [mh ^crocus] OR [mh ^crotalaria] OR [mh ^croton] OR [mh ^curcuma] OR [mh ^cycas] OR [mh ^cyperus] OR [mh ^"datura metel"] OR [mh ^"datura stramonium"] OR [mh ^derris] OR [mh ^dioclea] OR [mh ^echinacea] OR [mh ^eleutherococcus] OR [mh ^embelia] OR [mh ^ephedra] OR [mh ^epimedium] OR [mh ^erigeron] OR [mh ^eriodictyon] OR [mh ^erysimum] OR [mh ^erythrina] OR [mh ^eucalyptus] OR [mh ^eupatorium] OR [mh ^euphorbia] OR [mh ^euphorbiaceae] OR [mh ^evodia] OR [mh ^fallopia] OR [mh ^ficus] OR [mh ^forsythia] OR [mh ^fucus] OR [mh ^"garcinia cambogia"] OR [mh ^"garcinia kola"] OR [mh ^"garcinia mangostana"] OR [mh ^gastrodia] OR [mh ^glycyrrhiza] OR [mh ^goniothalamus] OR [mh ^gynostemma] OR [mh ^hamamelis] OR [mh ^harpagophytum] OR [mh ^heliotropium] OR [mh ^hemidesmus] OR [mh ^hibiscus] OR [mh ^hippophae] OR [mh ^houttuynia] OR [mh ^humulus] OR [mh ^hydrastis] OR [mh ^hydrocharitaceae] OR [mh ^hymenaea] OR [mh ^hyoscyamus] OR [mh ilex] OR [mh ^illicium] OR [mh ^inula] OR [mh ^juglans] OR [mh ^juniperus] OR [mh ^justicia] OR [mh ^"karaya gum"] OR [mh ^kava] OR [mh ^lantana] OR [mh ^laurus] OR [mh ^lavandula] OR [mh ^"lawsonia plant"] OR [mh ^leuzea] OR [mh ^ligusticum] OR [mh ^lithospermum] OR [mh ^lobelia] OR [mh ^luffa] OR [mh ^"mallotus plant"] OR [mh ^mandragora] OR [mh ^marrubium] OR [mh ^marsdenia] OR [mh ^matricaria] OR [mh ^maytenus] OR [mh ^"melia azedarach"] OR [mh ^melilotus] OR [mh ^"melinis repens"] OR [mh ^melissa] OR [mh ^mentha] OR [mh ^momordica] OR [mh ^monarda] OR [mh ^morinda] OR [mh ^"moringa oleifera"] OR [mh ^murraya] OR [mh ^myroxylon] OR [mh ^myrtus] OR [mh ^nelumbo] OR [mh ^nepeta] OR [mh ^ochrosia] OR [mh ^ocimum] OR [mh ^"ocimum sanctum"] OR [mh ^oenothera] OR [mh ^oplopanax] OR [mh ^equisetum] OR [mh ^pongamia] OR [mh ^"tanacetum parthenium"] OR [mh ^xanthium] OR [mh ^origanum] OR [mh ^orthosiphon] OR [mh ^paeonia] OR [mh panax] OR [mh ^papaver] OR [mh ^paris] OR [mh ^"parthenium hysterophorus"] OR [mh ^paullinia] OR [mh ^peganum] OR [mh ^pelargonium] OR [mh ^perilla] OR [mh ^peumus] OR [mh ^phaseolus] OR [mh ^phoradendron] OR [mh ^phyllanthus] OR [mh ^physostigma] OR [mh ^"phytolacca dodecandra"] OR [mh ^picrorhiza] OR [mh ^pilocarpus] OR [mh ^pimenta] OR [mh ^pinellia] OR [mh ^piper] OR [mh ^"piper betle"] OR [mh ^pistacia] OR [mh ^plantago] OR [mh ^plumbaginaceae] OR [mh ^podophyllum] OR [mh ^polyalthia] OR [mh ^polygonum] OR [mh ^primula] OR [mh ^prosopis] OR [mh ^psacalium] OR [mh ^pulicaria] OR [mh ^pulsatilla] OR [mh ^quillaja] OR [mh ^rauwolfia] OR [mh ^rhamnus] OR [mh ^rhodiola] OR [mh ^ribes] OR [mh ^"ricinus communis"] OR [mh ^rosa] OR [mh ^rubia] OR [mh ^rubus] OR [mh ^salacia] OR [mh ^salix] OR [mh ^salvia] OR [mh ^sambucus] OR [mh ^sanguinaria] OR [mh ^sanguisorba] OR [mh ^saponaria] OR [mh ^sassafras] OR [mh ^saussurea] OR [mh ^schisandra] OR [mh ^scopolia] OR [mh ^scrophularia] OR [mh ^scutellaria] OR [mh ^senecio] OR [mh ^sennosides] OR [mh ^serenoa] OR [mh ^silene] OR [mh ^"silybum marianum"] OR [mh ^smilax] OR [mh ^"solanum nigrum"] OR [mh ^"stephania tetrandra"] OR [mh ^sterculia] OR [mh ^stevia] OR [mh ^strophanthus] OR [mh ^strychnos] OR [mh ^swertia] OR [mh ^syzygium] OR [mh ^tabebuia] OR [mh ^tabernaemontana] OR [mh ^tanacetum] OR [mh ^taraxacum] OR [mh ^taxus] OR [mh ^terminalia] OR [mh ^thapsia] OR [mh ^tinospora] OR [mh ^toxicodendron] OR [mh ^tribulus] OR [mh ^trichosanthes] OR [mh ^trifolium] OR [mh ^trigonella] OR [mh ^tripterygium] OR [mh ^ulex] OR [mh ^uncaria] OR [mh ^"urtica dioica"] OR [mh ^"vaccinium myrtillus"] OR [mh ^valerian] OR [mh ^veratrum] OR [mh ^"veratrum alkaloids"] OR [mh ^verbascum] OR [mh ^vinca] OR [mh ^"viscum album"] OR [mh ^vitex] OR [mh ^vitis] OR [mh ^voacanga] OR [mh ^withania] OR [mh ^woodfordia] OR [mh ^yucca] OR [mh ^zanthoxylum] OR [mh ^"zingiber officinale"] \| 4389 \| \| #17 \| (abrus OR acacia OR acalypha OR achillea OR achyranthes OR aconite OR aconitum OR acorus OR "actaea racemosa" OR actinidia OR aegle OR aesculus OR agave OR ageratum OR agrimonia OR agrimony OR alchemilla OR "alchornea cordifolia" OR alfalfa OR almond OR aloe OR alpinia OR amaryllidaceae OR ambrosia OR ammi OR amsinckia OR amsonia OR anacardium OR ananas OR "andrographis paniculata" OR anemarrhena OR angelica OR anise OR annona OR anthemis OR apium OR apocynum OR apple OR apricot OR arachis OR aralia OR arctium OR arctostaphylos OR areca OR argemone OR aristolochia OR armoracia OR arnica OR artemisia OR artichoke OR asarum OR asimina OR asparagus OR aspidosperma OR "aster plant" OR astragalus OR atropa OR avocado OR bacopa OR ballota OR barley OR basil OR "bassia scoparia" OR bayberry OR beet OR belladonna OR berberis OR "beta vulgaris" OR "betel palm" OR bidens OR bilberry OR "bistorta officinalis" OR "bixa orellana" OR bixaceae OR "black cohosh" OR "black currant" OR blackberry OR blueberry OR "bonamia angiosperm" OR "bonamia plant" OR borago OR boswellia OR brassica OR broccoli OR bryonia OR buckthorn OR butterbur OR cabbage OR cacao OR caesalpinia OR calendula OR calophyllum OR calotropis OR "camellia sinensis" OR camphor OR camptotheca OR cananga OR cannabis OR capsella OR capsicum OR carica OR carrot OR cascara OR cashew OR cassia OR (cat* NEXT "claw") OR catha OR catharanthus OR catnip OR caulophyllum OR "cayenne pepper" OR celery OR centaurium OR "centella asiatica" OR cephaelis OR chamaemelum OR chamomile OR chaparral OR chelidonium OR chenopodium OR chive OR "chlorophytum borivilianum" OR chrysanthemum OR cicuta OR cimicifuga OR cinchona OR cinnamomum OR cinnamon OR cissus OR cistus OR citrus OR clitoria OR cnidium OR coca OR coconut OR cocos OR codonopsis OR coffee OR coix OR colchicum OR combretum OR comfrey OR commiphora OR condurango OR conium OR convallaria OR "coptis chinensis" OR corn OR costus OR cranberry OR crataegus OR crocus OR crotalaria OR croton OR cucurbita OR curcuma OR cycas OR cymbopogon OR "cynara scolymus" OR cyperus OR dandelion OR "datura metel" OR "daucus carota" OR derris OR "devil's claw" OR dioclea OR dioscorea OR "dong quai" OR "ecballium elaterium" OR echinacea OR elderberry OR eleutherococcus OR embelia OR ephedra OR epimedium OR equisetum OR "eremophila angiosperm" OR erigeron OR eriodictyon OR erysimum OR erythrina OR eucalyptus OR eupatorium OR euphorbia OR euphorbiaceae OR euterpe OR "evening primrose" OR evodia OR fallopia OR fennel OR fenugreek OR feverfew OR ficus OR fig OR flax OR flaxseed OR foeniculum OR forsythia OR fragaria OR frangula OR fucus OR "garcinia cambogia" OR "garcinia kola" OR "garcinia mangostana" OR gastrodia OR ginger OR "ginkgo biloba" OR ginseng OR "glycine max" OR glycyrrhiza OR goldenseal OR goniothalamus OR grape OR grapefruit OR "green tea" OR guarana OR guava OR gynostemma OR hamamelis OR harpagophytum OR hawthorn OR heliotropium OR hemidesmus OR hibiscus OR hippophae OR hops OR hordeum OR "horse chestnut" OR horseradish OR houttuynia OR humulus OR hydrastis OR hydrocharitaceae OR hymenaea OR hyoscyamus OR ilex OR illicium OR inula OR ivy OR juglans OR juniper OR juniperus OR justicia OR "karaya gum" OR kava OR kiwi OR "kochia scoparia" OR (lady* NEXT "mantle") OR "lagenaria siceraria" OR lantana OR laurus OR lavandula OR lavender OR "lawsonia inermis" OR "lawsonia plant" OR lemon OR leuzea OR licorice OR ligusticum OR "lily of the valley" OR lime OR lithospermum OR lobelia OR luffa OR "macrotyloma uniflorum" OR "mallotus philippinensis" OR "mallotus plant" OR malus OR mandragora OR marigold OR "maritime squill" OR marrubium OR marsdenia OR matricaria OR maytenus OR "medicago sativa" OR melaleuca OR "melia azedarach" OR melilotus OR "melinis repens" OR melissa OR mentha OR "milk thistle" OR mint OR mistletoe OR momordica OR monarda OR morinda OR "moringa oleifera" OR mugwort OR murraya OR mustard OR myrica OR myristica OR myroxylon OR myrtle OR myrtus OR nelumbo OR nepeta OR nerium OR noni OR nutmeg OR oat OR ochrosia OR ocimum OR oenothera OR olea OR oleander OR olive OR onions OR oplopanax OR oregano OR origanum OR oroxylum OR orthosiphon OR paeonia OR panax OR papaver OR papaya OR paris OR parsley OR parsnip OR "parthenium hysterophorus" OR passiflora OR passionflower OR pastinaca OR paullinia OR pausinystalia OR peach OR peanut OR pear OR peganum OR pelargonium OR pennyroyal OR perilla OR persea OR petasites OR petroselinum OR peumus OR phaseolus OR phoradendron OR phyllanthus OR physostigma OR "phytolacca dodecandra" OR picrorhiza OR pilocarpus OR pimenta OR pineapple OR pinellia OR piper OR pistacia OR plantago OR plumbaginaceae OR "plumbago zeylanica" OR podophyllum OR "poison ivy" OR polyalthia OR polygonum OR pomegranate OR pongamia OR potato OR primula OR prosopis OR prunus OR psacalium OR psidium OR psyllium OR pulicaria OR pulsatilla OR pumpkin OR pygeum OR pyrus OR quillaja OR radish OR ragwort OR raphanus OR raspberry OR rauvolfia OR rauwolfia OR "red clover" OR rhamnus OR rheum OR rhodiola OR rhubarb OR ribes OR "ricinus communis" OR rosa OR rose OR rosemary OR rubia OR rubus OR sabal OR sage OR salacia OR salix OR salvia OR sambucus OR sanguinaria OR sanguisorba OR saponaria OR sarsaparilla OR sassafras OR saussurea OR "saw palmetto" OR schinus OR schisandra OR scopolia OR scrophularia OR scutellaria OR senecio OR "senegalia catechu" OR "senegalia modesta" OR "senegalia senegal" OR senna OR sennosides OR serenoa OR silene OR "silybum marianum" OR "slippery elm" OR "smallanthus sonchifolius" OR smilax OR "solanum lycopersicum" OR "solanum nigrum" OR "solanum tuberosum" OR "sour cherry" OR soybeans OR spinach OR "spinacia oleracea" OR squash OR "stephania tetrandra" OR sterculia OR stevia OR "stinging nettle" OR strawberry OR strophanthus OR strychnos OR swertia OR syzygium OR tabebuia OR tabernaemontana OR talus OR tanacetum OR taraxacum OR taxus OR "tea tree" OR terminalia OR thapsia OR thyme OR "thymus plant" OR tinospora OR tobacco OR tomatoes OR toxicodendron OR tribulus OR trichosanthes OR "tridax procumbens" OR trifolium OR trigonella OR tripterygium OR turmeric OR ulex OR ulmus OR uncaria OR "urtica dioica" OR "vaccinium macrocarpon" OR "vaccinium myrtillus" OR "vachellia farnesiana" OR "vachellia nilotica" OR "vachellia tortilis" OR valerian OR valeriana OR veratrum OR verbascum OR vinca OR "viscum album" OR vitex OR vitis OR voacanga OR "wild cherry" OR "wild yam" OR willow OR "witch hazel" OR withania OR woodfordia OR "wrightia tinctoria" OR "wrightia tomentosa" OR xanthium OR xanthorrhiza OR yarrow OR yew OR yohimbe OR yucca OR zanthoxylum OR "zataria multiflora" OR "zea mays" OR "zingiber officinale" ):ti,ab,kw \| 72157 \| \| #18 \| #16 OR #17 \| 72157 \| \| #19 \| #12 OR #15 OR #18 \| 96893 \| \| #20 \| [mh ^"Trigeminal Neuralgia"] \| 234 \| \| #21 \| [mh ^"Trigeminal Nerve Diseases"] \| 18 \| \| #22 \| (("drigeminal" NEXT neuropath*) OR ("epileptiform" NEXT neuralg*) OR ("fothergill" NEXT disease*) OR prosopalgia OR "tic doloureux" OR "tic douloureux" OR ("trifacial" NEXT neuralg*) OR (trigemin* NEXT neuralg*) OR (trigemin* NEXT "nerve" NEXT disease*) OR (trigemin* NEXT "nerve neuralgia") OR (trigemin* NEXT "nerve" NEXT disorder*) OR (trigemin* NEXT "neuropathic pain") ):ti,ab,kw \| 592 \| \| #23 \| ((((trigemin* NEXT "nerve") OR (mandibul* NEXT "nerve") OR (mandibul* NEXT "neuropathic") OR (maxilla* NEXT "nerve") OR "chronic neuralgia" ) AND pain )):ti,ab,kw \| 685 \| \| #24 \| #20 OR #21 OR #22 OR #23 \| 1168 \| \| #25 \| #19 AND #24 \| 149 \| | |

4. Web of Science Core Collection

| Interface: Clarivate Analytics  Editions = A&HCI , ESCI , SCI-EXPANDED , SSCI  Date of Search: 21 March 2024  Number of hits: 812 | Field labels   - TS/Topic = title, abstract, author keywords and Keywords Plus - NEAR/x = within x words, regardless of order - * = truncation of word for alternate endings   Note: the *Exact search*-function was used for all the searches |
| --- | --- |
| \| # \| Search Query \| Results \| \| --- \| --- \| --- \| \| 1 \| TS=("botanical medicin*" OR ethno-medicin* OR ethnobotan* OR ethnomedicin* OR "folk medicin**" OR "folk remed*" OR "healing plant*" OR herb OR herbaceous OR herbal OR herbalism OR herbs OR "home remed*" OR kampo OR kanpo OR "medicinal plant*" OR "pharmaceutical plant*" OR pharmacognos* OR phyto-medic* OR phytomedic* OR phytotherap* OR "plant-based medicin*" OR "plant-based remedy" OR "plant extract*" OR "plant medicin*" OR "plant oil$" OR "primitive medicin*" OR saiko-keishi-to OR "traditional medicin*" OR uyakujunkisan OR "vegetable oil$" ) \| 267974 \| \| 2 \| TS=("allium sativum" OR amizepine OR axsain OR "boletus lucidus" OR chili OR capsaicin OR capsaicine OR capsicum OR capsidol OR capsin OR capzasin OR carbamazepine OR carbazepin OR camomil* OR chamomil* OR corydalis OR engosaku OR epitol OR finlepsin OR garlic$ OR gelcen OR "ganoderma lucidum" OR hyeonhosaek OR hypericum$ OR katrum OR kudzu$ OR lingzhi OR matricaria OR neurotol OR "pachyrhizus thunbergianus" OR pepper$ OR plamatine OR pueraria$ OR puerariae$ OR reishi OR "st. john* wort" OR "st. johnswort" OR "saint johnswort" OR "saint john* wort" OR tegretol OR "yan hu suo" OR yanhusuo OR zacin OR zostrix ) \| 109019 \| \| 3 \| TS=(abrus OR acacia OR acalypha OR achillea OR achyranthes OR aconite OR aconitum OR acorus OR "actaea racemosa" OR actinidia OR aegle OR aesculus OR agave OR ageratum OR agrimonia OR agrimony OR alchemilla OR "alchornea cordifolia" OR alfalfa OR almond OR aloe OR alpinia OR amaryllidaceae OR ambrosia OR ammi OR amsinckia OR amsonia OR anacardium OR ananas OR "andrographis paniculata" OR anemarrhena OR angelica OR anise OR annona OR anthemis OR apium OR apocynum OR apple OR apricot OR arachis OR aralia OR arctium OR arctostaphylos OR areca OR argemone OR aristolochia OR armoracia OR arnica OR artemisia OR artichoke OR asarum OR asimina OR asparagus OR aspidosperma OR "aster plant" OR astragalus OR atropa OR avocado OR bacopa OR ballota OR barley OR basil OR "bassia scoparia" OR bayberry OR beet OR belladonna OR berberis OR "beta vulgaris" OR "betel palm" OR bidens OR bilberry OR "bistorta officinalis" OR "bixa orellana" OR bixaceae OR "black cohosh" OR "black currant" OR blackberry OR blueberry OR "bonamia angiosperm" OR "bonamia plant" OR borago OR boswellia OR brassica OR broccoli OR bryonia OR buckthorn OR butterbur OR cabbage OR cacao OR caesalpinia OR calendula OR calophyllum OR calotropis OR "camellia sinensis" OR camphor OR camptotheca OR cananga OR cannabis OR capsella OR capsicum OR carica OR carrot OR cascara OR cashew OR cassia OR "cat* claw" OR catha OR catharanthus OR catnip OR caulophyllum OR "cayenne pepper" OR celery OR centaurium OR "centella asiatica" OR cephaelis OR chamaemelum OR chamomile OR chaparral OR chelidonium OR chenopodium OR chive OR "chlorophytum borivilianum" OR chrysanthemum OR cicuta OR cimicifuga OR cinchona OR cinnamomum OR cinnamon OR cissus OR cistus OR citrus OR clitoria OR cnidium OR coca OR coconut OR cocos OR codonopsis OR coffee OR coix OR colchicum OR combretum OR comfrey OR commiphora OR condurango OR conium OR convallaria OR "coptis chinensis" OR corn OR costus OR cranberry OR crataegus OR crocus OR crotalaria OR croton OR cucurbita OR curcuma OR cycas OR cymbopogon OR "cynara scolymus" OR cyperus OR dandelion OR "datura metel" OR "daucus carota" OR derris OR "devil's claw" OR dioclea OR dioscorea OR "dong quai" OR "ecballium elaterium" OR echinacea OR elderberry OR eleutherococcus OR embelia OR ephedra OR epimedium OR equisetum OR "eremophila angiosperm" OR erigeron OR eriodictyon OR erysimum OR erythrina OR eucalyptus OR eupatorium OR euphorbia OR euphorbiaceae OR euterpe OR "evening primrose" OR evodia OR fallopia OR fennel OR fenugreek OR feverfew OR ficus OR fig OR flax OR flaxseed OR foeniculum OR forsythia OR fragaria OR frangula OR fucus OR "garcinia cambogia" OR "garcinia kola" OR "garcinia mangostana" OR gastrodia OR ginger OR "ginkgo biloba" OR ginseng OR "glycine max" OR glycyrrhiza OR goldenseal OR goniothalamus OR grape OR grapefruit OR "green tea" OR guarana OR guava OR gynostemma OR hamamelis OR harpagophytum OR hawthorn OR heliotropium OR hemidesmus OR hibiscus OR hippophae OR hops OR hordeum OR "horse chestnut" OR horseradish OR houttuynia OR humulus OR hydrastis OR hydrocharitaceae OR hymenaea OR hyoscyamus OR ilex OR illicium OR inula OR ivy OR juglans OR juniper OR juniperus OR justicia OR "karaya gum" OR kava OR kiwi OR "kochia scoparia" OR "lady* mantle" OR "lagenaria siceraria" OR lantana OR laurus OR lavandula OR lavender OR "lawsonia inermis" OR "lawsonia plant" OR lemon OR leuzea OR licorice OR ligusticum OR "lily of the valley" OR lime OR lithospermum OR lobelia OR luffa OR "macrotyloma uniflorum" OR "mallotus philippinensis" OR "mallotus plant" OR malus OR mandragora OR marigold OR "maritime squill" OR marrubium OR marsdenia OR matricaria OR maytenus OR "medicago sativa" OR melaleuca OR "melia azedarach" OR melilotus OR "melinis repens" OR melissa OR mentha OR "milk thistle" OR mint OR mistletoe OR momordica OR monarda OR morinda OR "moringa oleifera" OR mugwort OR murraya OR mustard OR myrica OR myristica OR myroxylon OR myrtle OR myrtus OR nelumbo OR nepeta OR nerium OR noni OR nutmeg OR oat OR ochrosia OR ocimum OR oenothera OR olea OR oleander OR olive OR onions OR oplopanax OR oregano OR origanum OR oroxylum OR orthosiphon OR paeonia OR panax OR papaver OR papaya OR paris OR parsley OR parsnip OR "parthenium hysterophorus" OR passiflora OR passionflower OR pastinaca OR paullinia OR pausinystalia OR peach OR peanut OR pear OR peganum OR pelargonium OR pennyroyal OR perilla OR persea OR petasites OR petroselinum OR peumus OR phaseolus OR phoradendron OR phyllanthus OR physostigma OR "phytolacca dodecandra" OR picrorhiza OR pilocarpus OR pimenta OR pineapple OR pinellia OR piper OR pistacia OR plantago OR plumbaginaceae OR "plumbago zeylanica" OR podophyllum OR "poison ivy" OR polyalthia OR polygonum OR pomegranate OR pongamia OR potato OR primula OR prosopis OR prunus OR psacalium OR psidium OR psyllium OR pulicaria OR pulsatilla OR pumpkin OR pygeum OR pyrus OR quillaja OR radish OR ragwort OR raphanus OR raspberry OR rauvolfia OR rauwolfia OR "red clover" OR rhamnus OR rheum OR rhodiola OR rhubarb OR ribes OR "ricinus communis" OR rosa OR rose OR rosemary OR rubia OR rubus OR sabal OR sage OR salacia OR salix OR salvia OR sambucus OR sanguinaria OR sanguisorba OR saponaria OR sarsaparilla OR sassafras OR saussurea OR "saw palmetto" OR schinus OR schisandra OR scopolia OR scrophularia OR scutellaria OR senecio OR "senegalia catechu" OR "senegalia modesta" OR "senegalia senegal" OR senna OR sennosides OR serenoa OR silene OR "silybum marianum" OR "slippery elm" OR "smallanthus sonchifolius" OR smilax OR "solanum lycopersicum" OR "solanum nigrum" OR "solanum tuberosum" OR "sour cherry" OR soybeans OR spinach OR "spinacia oleracea" OR squash OR "stephania tetrandra" OR sterculia OR stevia OR "stinging nettle" OR strawberry OR strophanthus OR strychnos OR swertia OR syzygium OR tabebuia OR tabernaemontana OR talus OR tanacetum OR taraxacum OR taxus OR "tea tree" OR terminalia OR thapsia OR thyme OR "thymus plant" OR tinospora OR tobacco OR tomatoes OR toxicodendron OR tribulus OR trichosanthes OR "tridax procumbens" OR trifolium OR trigonella OR tripterygium OR turmeric OR ulex OR ulmus OR uncaria OR "urtica dioica" OR "vaccinium macrocarpon" OR "vaccinium myrtillus" OR "vachellia farnesiana" OR "vachellia nilotica" OR "vachellia tortilis" OR valerian OR valeriana OR veratrum OR verbascum OR vinca OR "viscum album" OR vitex OR vitis OR voacanga OR "wild cherry" OR "wild yam" OR willow OR "witch hazel" OR withania OR woodfordia OR "wrightia tinctoria" OR "wrightia tomentosa" OR xanthium OR xanthorrhiza OR yarrow OR yew OR yohimbe OR yucca OR zanthoxylum OR "zataria multiflora" OR "zea mays" OR "zingiber officinale" ) \| 2018267 \| \| 4 \| #1 OR #2 OR #3 \| 2247760 \| \| 5 \| TS=("drigeminal neuropath*" OR "epileptiform neuralg*" OR "fothergill disease*" OR prosopalgia OR "tic doloureux" OR "tic douloureux" OR "trifacial neuralg*" OR "trigemin* neuralg*" OR "trigemin* nerve disease*" OR "trigemin* nerve neuralgia" OR "trigemin* nerve disorder*" OR "trigemin* neuropathic pain" ) \| 8133 \| \| 6 \| TS=(("trigemin* nerve" OR "mandibul* nerve" OR "mandibul* neuropathic" OR "maxilla* nerve" OR "chronic neuralgia" ) AND pain ) \| 2519 \| \| 7 \| #5 OR #6 \| 9501 \| \| 8 \| #4 AND #7 \| 812 \| | |

6. Cinahl

| Interface: Ebsco  Date of Search: 21 March 2024  Number of hits: 272 | Field labels   - MH+ = exploded Cinahl Heading - MH = non exploded Cinahl Heading - TI = title - AB = abstract - Nx = within x words, regardless of order - * = truncation of word for alternate endings   Note: sometimes “quotation marks” are needed for single search terms to avoid automatic term mapping (lemmatization)1 |
| --- | --- |
| \| # \| Query \| Results \| \| --- \| --- \| --- \| \| S1 \| (MH "Alternative Therapies") \| 39,967 \| \| S2 \| (MH "Drugs, Chinese Herbal") \| 11,453 \| \| S3 \| (MH "Medicine, Herbal") \| 14,825 \| \| S4 \| (MH "Medicine, Traditional") \| 6,806 \| \| S5 \| (MH "Plant Extracts") \| 41,397 \| \| S6 \| (MH "Plant Extracts") \| 41,397 \| \| S7 \| (MH "Plant Oils") \| 4,500 \| \| S8 \| (MH "Plants, Medicinal") \| 33,151 \| \| S9 \| TI(("alternative medicin*" OR "alternative therap*" OR "botanical medicin*" OR "complementary therap*" OR "complementary medicin*" OR ethno-medicin* OR ethnobotan* OR ethnomedicin* OR "folk medicin*" OR "folk remed*" OR "healing plant*" OR herb OR herbaceous OR herbal OR herbalism OR herbs OR "home remed*" OR kampo OR kanpo OR "medicinal plant*" OR "pharmaceutical plant*" OR pharmacognos* OR phyto-medic* OR phytomedic* OR phytotherap* OR "plant-based medicin*" OR "plant-based remedy" OR "plant extract*" OR "plant medicin*" OR "plant oil#" OR "primitive medicin*" OR saiko-keishi-to OR "traditional medicin*" OR uyakujunkisan OR "vegetable oil#" )) OR AB(("alternative medicin*" OR "alternative therap*" OR "botanical medicin*" OR "complementary therap*" OR "complementary medicin*" OR ethno-medicin* OR ethnobotan* OR ethnomedicin* OR "folk medicin*" OR "folk remed*" OR "healing plant*" OR herb OR herbaceous OR herbal OR herbalism OR herbs OR "home remed*" OR kampo OR kanpo OR "medicinal plant*" OR "pharmaceutical plant*" OR pharmacognos* OR phyto-medic* OR phytomedic* OR phytotherap* OR "plant-based medicin*" OR "plant-based remedy" OR "plant extract*" OR "plant medicin*" OR "plant oil#" OR "primitive medicin*" OR saiko-keishi-to OR "traditional medicin*" OR uyakujunkisan OR "vegetable oil#")) \| 53,958 \| \| S10 \| S1 OR S2 OR S3 OR S4 OR S5 OR S6 OR S7 OR S8 OR S9 \| 126,116 \| \| S11 \| (MH "Capsaicin") OR (MH "Carbamazepine") \| 3,350 \| \| S12 \| TI(("allium sativum" OR amizepine OR axsain OR "boletus lucidus" OR chili OR capsaicin OR capsaicine OR capsicum OR capsidol OR capsin OR capzasin OR carbamazepine OR carbazepin OR camomil* OR chamomil* OR corydalis OR engosaku OR epitol OR finlepsin OR garlic# OR gelcen OR "ganoderma lucidum" OR hyeonhosaek OR hypericum# OR katrum OR kudzu# OR lingzhi OR matricaria OR neurotol OR "pachyrhizus thunbergianus" OR pepper# OR plamatine OR pueraria# OR puerariae# OR reishi OR "st. john* wort" OR "st. johnswort" OR "saint john* wort" OR "saint johnswort" OR tegretol OR "yan hu suo" OR yanhusuo OR zacin OR zostrix )) OR AB(("allium sativum" OR amizepine OR axsain OR "boletus lucidus" OR chili OR capsaicin OR capsaicine OR capsicum OR capsidol OR capsin OR capzasin OR carbamazepine OR carbazepin OR camomil* OR chamomil* OR corydalis OR engosaku OR epitol OR finlepsin OR garlic# OR gelcen OR "ganoderma lucidum" OR hyeonhosaek OR hypericum# OR katrum OR kudzu# OR lingzhi OR matricaria OR neurotol OR "pachyrhizus thunbergianus" OR pepper# OR plamatine OR pueraria# OR puerariae# OR reishi OR "st. john* wort" OR "st. johnswort" OR "saint john* wort" OR "saint johnswort" OR tegretol OR "yan hu suo" OR yanhusuo OR zacin OR zostrix )) \| 8,331 \| \| S13 \| S11 OR S12 \| 9,597 \| \| S14 \| (MH "Plants, Medicinal+") \| 92,523 \| \| S15 \| TI((abrus OR acacia OR acalypha OR achillea OR achyranthes OR aconite OR aconitum OR acorus OR "actaea racemosa" OR actinidia OR aegle OR aesculus OR agave OR ageratum OR agrimonia OR agrimony OR alchemilla OR "alchornea cordifolia" OR alfalfa OR almond OR aloe OR alpinia OR amaryllidaceae OR ambrosia OR ammi OR amsinckia OR amsonia OR anacardium OR ananas OR "andrographis paniculata" OR anemarrhena OR angelica OR anise OR annona OR anthemis OR apium OR apocynum OR apple OR apricot OR arachis OR aralia OR arctium OR arctostaphylos OR areca OR argemone OR aristolochia OR armoracia OR arnica OR artemisia OR artichoke OR asarum OR asimina OR asparagus OR aspidosperma OR "aster plant" OR astragalus OR atropa OR avocado OR bacopa OR ballota OR barley OR basil OR "bassia scoparia" OR bayberry OR beet OR belladonna OR berberis OR "beta vulgaris" OR "betel palm" OR bidens OR bilberry OR "bistorta officinalis" OR "bixa orellana" OR bixaceae OR "black cohosh" OR "black currant" OR blackberry OR blueberry OR "bonamia angiosperm" OR "bonamia plant" OR borago OR boswellia OR brassica OR broccoli OR bryonia OR buckthorn OR butterbur OR cabbage OR cacao OR caesalpinia OR calendula OR calophyllum OR calotropis OR "camellia sinensis" OR camphor OR camptotheca OR cananga OR cannabis OR capsella OR capsicum OR carica OR carrot OR cascara OR cashew OR cassia OR "cat* claw" OR catha OR catharanthus OR catnip OR caulophyllum OR "cayenne pepper" OR celery OR centaurium OR "centella asiatica" OR cephaelis OR chamaemelum OR chamomile OR chaparral OR chelidonium OR chenopodium OR chive OR "chlorophytum borivilianum" OR chrysanthemum OR cicuta OR cimicifuga OR cinchona OR cinnamomum OR cinnamon OR cissus OR cistus OR citrus OR clitoria OR cnidium OR coca OR coconut OR cocos OR codonopsis OR coffee OR coix OR colchicum OR combretum OR comfrey OR commiphora OR condurango OR conium OR convallaria OR "coptis chinensis" OR corn OR costus OR cranberry OR crataegus OR crocus OR crotalaria OR croton OR cucurbita OR curcuma OR cycas OR cymbopogon OR "cynara scolymus" OR cyperus OR dandelion OR "datura metel" OR "daucus carota" OR derris OR "devil's claw" OR dioclea OR dioscorea OR "dong quai" OR "ecballium elaterium" OR echinacea OR elderberry OR eleutherococcus OR embelia OR ephedra OR epimedium OR equisetum OR "eremophila angiosperm" OR erigeron OR eriodictyon OR erysimum OR erythrina OR eucalyptus OR eupatorium OR euphorbia OR euphorbiaceae OR euterpe OR "evening primrose" OR evodia OR fallopia OR fennel OR fenugreek OR feverfew OR ficus OR fig OR flax OR flaxseed OR foeniculum OR forsythia OR fragaria OR frangula OR fucus OR "garcinia cambogia" OR "garcinia kola" OR "garcinia mangostana" OR gastrodia OR ginger OR "ginkgo biloba" OR ginseng OR "glycine max" OR glycyrrhiza OR goldenseal OR goniothalamus OR grape OR grapefruit OR "green tea" OR guarana OR guava OR gynostemma OR hamamelis OR harpagophytum OR hawthorn OR heliotropium OR hemidesmus OR hibiscus OR hippophae OR hops OR hordeum OR "horse chestnut" OR horseradish OR houttuynia OR humulus OR hydrastis OR hydrocharitaceae OR hymenaea OR hyoscyamus OR ilex OR illicium OR inula OR ivy OR juglans OR juniper OR juniperus OR justicia OR "karaya gum" OR kava OR kiwi OR "kochia scoparia" OR "lady* mantle" OR "lagenaria siceraria" OR lantana OR laurus OR lavandula OR lavender OR "lawsonia inermis" OR "lawsonia plant" OR lemon OR leuzea OR licorice OR ligusticum OR "lily of the valley" OR lime OR lithospermum OR lobelia OR luffa OR "macrotyloma uniflorum" OR "mallotus philippinensis" OR "mallotus plant" OR malus OR mandragora OR marigold OR "maritime squill" OR marrubium OR marsdenia OR matricaria OR maytenus OR "medicago sativa" OR melaleuca OR "melia azedarach" OR melilotus OR "melinis repens" OR melissa OR mentha OR "milk thistle" OR mint OR mistletoe OR momordica OR monarda OR morinda OR "moringa oleifera" OR mugwort OR murraya OR mustard OR myrica OR myristica OR myroxylon OR myrtle OR myrtus OR nelumbo OR nepeta OR nerium OR noni OR nutmeg OR oat OR ochrosia OR ocimum OR oenothera OR olea OR oleander OR olive OR onions OR oplopanax OR oregano OR origanum OR oroxylum OR orthosiphon OR paeonia OR panax OR papaver OR papaya OR paris OR parsley OR parsnip OR "parthenium hysterophorus" OR passiflora OR passionflower OR pastinaca OR paullinia OR pausinystalia OR peach OR peanut OR pear OR peganum OR pelargonium OR pennyroyal OR perilla OR persea OR petasites OR petroselinum OR peumus OR phaseolus OR phoradendron OR phyllanthus OR physostigma OR "phytolacca dodecandra" OR picrorhiza OR pilocarpus OR pimenta OR pineapple OR pinellia OR piper OR pistacia OR plantago OR plumbaginaceae OR "plumbago zeylanica" OR podophyllum OR "poison ivy" OR polyalthia OR polygonum OR pomegranate OR pongamia OR potato OR primula OR prosopis OR prunus OR psacalium OR psidium OR psyllium OR pulicaria OR pulsatilla OR pumpkin OR pygeum OR pyrus OR quillaja OR radish OR ragwort OR raphanus OR raspberry OR rauvolfia OR rauwolfia OR "red clover" OR rhamnus OR rheum OR rhodiola OR rhubarb OR ribes OR "ricinus communis" OR rosa OR rose OR rosemary OR rubia OR rubus OR sabal OR sage OR salacia OR salix OR salvia OR sambucus OR sanguinaria OR sanguisorba OR saponaria OR sarsaparilla OR sassafras OR saussurea OR "saw palmetto" OR schinus OR schisandra OR scopolia OR scrophularia OR scutellaria OR senecio OR "senegalia catechu" OR "senegalia modesta" OR "senegalia senegal" OR senna OR sennosides OR serenoa OR silene OR "silybum marianum" OR "slippery elm" OR "smallanthus sonchifolius" OR smilax OR "solanum lycopersicum" OR "solanum nigrum" OR "solanum tuberosum" OR "sour cherry" OR soybeans OR spinach OR "spinacia oleracea" OR squash OR "stephania tetrandra" OR sterculia OR stevia OR "stinging nettle" OR strawberry OR strophanthus OR strychnos OR swertia OR syzygium OR tabebuia OR tabernaemontana OR talus OR tanacetum OR taraxacum OR taxus OR "tea tree" OR terminalia OR thapsia OR thyme OR "thymus plant" OR tinospora OR tobacco OR tomatoes OR toxicodendron OR tribulus OR trichosanthes OR "tridax procumbens" OR trifolium OR trigonella OR tripterygium OR turmeric OR ulex OR ulmus OR uncaria OR "urtica dioica" OR "vaccinium macrocarpon" OR "vaccinium myrtillus" OR "vachellia farnesiana" OR "vachellia nilotica" OR "vachellia tortilis" OR valerian OR valeriana OR veratrum OR verbascum OR vinca OR "viscum album" OR vitex OR vitis OR voacanga OR "wild cherry" OR "wild yam" OR willow OR "witch hazel" OR withania OR woodfordia OR "wrightia tinctoria" OR "wrightia tomentosa" OR xanthium OR xanthorrhiza OR yarrow OR yew OR yohimbe OR yucca OR zanthoxylum OR "zataria multiflora" OR "zea mays" OR "zingiber officinale" )) OR AB((abrus OR acacia OR acalypha OR achillea OR achyranthes OR aconite OR aconitum OR acorus OR "actaea racemosa" OR actinidia OR aegle OR aesculus OR agave OR ageratum OR agrimonia OR agrimony OR alchemilla OR "alchornea cordifolia" OR alfalfa OR almond OR aloe OR alpinia OR amaryllidaceae OR ambrosia OR ammi OR amsinckia OR amsonia OR anacardium OR ananas OR "andrographis paniculata" OR anemarrhena OR angelica OR anise OR annona OR anthemis OR apium OR apocynum OR apple OR apricot OR arachis OR aralia OR arctium OR arctostaphylos OR areca OR argemone OR aristolochia OR armoracia OR arnica OR artemisia OR artichoke OR asarum OR asimina OR asparagus OR aspidosperma OR "aster plant" OR astragalus OR atropa OR avocado OR bacopa OR ballota OR barley OR basil OR "bassia scoparia" OR bayberry OR beet OR belladonna OR berberis OR "beta vulgaris" OR "betel palm" OR bidens OR bilberry OR "bistorta officinalis" OR "bixa orellana" OR bixaceae OR "black cohosh" OR "black currant" OR blackberry OR blueberry OR "bonamia angiosperm" OR "bonamia plant" OR borago OR boswellia OR brassica OR broccoli OR bryonia OR buckthorn OR butterbur OR cabbage OR cacao OR caesalpinia OR calendula OR calophyllum OR calotropis OR "camellia sinensis" OR camphor OR camptotheca OR cananga OR cannabis OR capsella OR capsicum OR carica OR carrot OR cascara OR cashew OR cassia OR "cat* claw" OR catha OR catharanthus OR catnip OR caulophyllum OR "cayenne pepper" OR celery OR centaurium OR "centella asiatica" OR cephaelis OR chamaemelum OR chamomile OR chaparral OR chelidonium OR chenopodium OR chive OR "chlorophytum borivilianum" OR chrysanthemum OR cicuta OR cimicifuga OR cinchona OR cinnamomum OR cinnamon OR cissus OR cistus OR citrus OR clitoria OR cnidium OR coca OR coconut OR cocos OR codonopsis OR coffee OR coix OR colchicum OR combretum OR comfrey OR commiphora OR condurango OR conium OR convallaria OR "coptis chinensis" OR corn OR costus OR cranberry OR crataegus OR crocus OR crotalaria OR croton OR cucurbita OR curcuma OR cycas OR cymbopogon OR "cynara scolymus" OR cyperus OR dandelion OR "datura metel" OR "daucus carota" OR derris OR "devil's claw" OR dioclea OR dioscorea OR "dong quai" OR "ecballium elaterium" OR echinacea OR elderberry OR eleutherococcus OR embelia OR ephedra OR epimedium OR equisetum OR "eremophila angiosperm" OR erigeron OR eriodictyon OR erysimum OR erythrina OR eucalyptus OR eupatorium OR euphorbia OR euphorbiaceae OR euterpe OR "evening primrose" OR evodia OR fallopia OR fennel OR fenugreek OR feverfew OR ficus OR fig OR flax OR flaxseed OR foeniculum OR forsythia OR fragaria OR frangula OR fucus OR "garcinia cambogia" OR "garcinia kola" OR "garcinia mangostana" OR gastrodia OR ginger OR "ginkgo biloba" OR ginseng OR "glycine max" OR glycyrrhiza OR goldenseal OR goniothalamus OR grape OR grapefruit OR "green tea" OR guarana OR guava OR gynostemma OR hamamelis OR harpagophytum OR hawthorn OR heliotropium OR hemidesmus OR hibiscus OR hippophae OR hops OR hordeum OR "horse chestnut" OR horseradish OR houttuynia OR humulus OR hydrastis OR hydrocharitaceae OR hymenaea OR hyoscyamus OR ilex OR illicium OR inula OR ivy OR juglans OR juniper OR juniperus OR justicia OR "karaya gum" OR kava OR kiwi OR "kochia scoparia" OR "lady* mantle" OR "lagenaria siceraria" OR lantana OR laurus OR lavandula OR lavender OR "lawsonia inermis" OR "lawsonia plant" OR lemon OR leuzea OR licorice OR ligusticum OR "lily of the valley" OR lime OR lithospermum OR lobelia OR luffa OR "macrotyloma uniflorum" OR "mallotus philippinensis" OR "mallotus plant" OR malus OR mandragora OR marigold OR "maritime squill" OR marrubium OR marsdenia OR matricaria OR maytenus OR "medicago sativa" OR melaleuca OR "melia azedarach" OR melilotus OR "melinis repens" OR melissa OR mentha OR "milk thistle" OR mint OR mistletoe OR momordica OR monarda OR morinda OR "moringa oleifera" OR mugwort OR murraya OR mustard OR myrica OR myristica OR myroxylon OR myrtle OR myrtus OR nelumbo OR nepeta OR nerium OR noni OR nutmeg OR oat OR ochrosia OR ocimum OR oenothera OR olea OR oleander OR olive OR onions OR oplopanax OR oregano OR origanum OR oroxylum OR orthosiphon OR paeonia OR panax OR papaver OR papaya OR paris OR parsley OR parsnip OR "parthenium hysterophorus" OR passiflora OR passionflower OR pastinaca OR paullinia OR pausinystalia OR peach OR peanut OR pear OR peganum OR pelargonium OR pennyroyal OR perilla OR persea OR petasites OR petroselinum OR peumus OR phaseolus OR phoradendron OR phyllanthus OR physostigma OR "phytolacca dodecandra" OR picrorhiza OR pilocarpus OR pimenta OR pineapple OR pinellia OR piper OR pistacia OR plantago OR plumbaginaceae OR "plumbago zeylanica" OR podophyllum OR "poison ivy" OR polyalthia OR polygonum OR pomegranate OR pongamia OR potato OR primula OR prosopis OR prunus OR psacalium OR psidium OR psyllium OR pulicaria OR pulsatilla OR pumpkin OR pygeum OR pyrus OR quillaja OR radish OR ragwort OR raphanus OR raspberry OR rauvolfia OR rauwolfia OR "red clover" OR rhamnus OR rheum OR rhodiola OR rhubarb OR ribes OR "ricinus communis" OR rosa OR rose OR rosemary OR rubia OR rubus OR sabal OR sage OR salacia OR salix OR salvia OR sambucus OR sanguinaria OR sanguisorba OR saponaria OR sarsaparilla OR sassafras OR saussurea OR "saw palmetto" OR schinus OR schisandra OR scopolia OR scrophularia OR scutellaria OR senecio OR "senegalia catechu" OR "senegalia modesta" OR "senegalia senegal" OR senna OR sennosides OR serenoa OR silene OR "silybum marianum" OR "slippery elm" OR "smallanthus sonchifolius" OR smilax OR "solanum lycopersicum" OR "solanum nigrum" OR "solanum tuberosum" OR "sour cherry" OR soybeans OR spinach OR "spinacia oleracea" OR squash OR "stephania tetrandra" OR sterculia OR stevia OR "stinging nettle" OR strawberry OR strophanthus OR strychnos OR swertia OR syzygium OR tabebuia OR tabernaemontana OR talus OR tanacetum OR taraxacum OR taxus OR "tea tree" OR terminalia OR thapsia OR thyme OR "thymus plant" OR tinospora OR tobacco OR tomatoes OR toxicodendron OR tribulus OR trichosanthes OR "tridax procumbens" OR trifolium OR trigonella OR tripterygium OR turmeric OR ulex OR ulmus OR uncaria OR "urtica dioica" OR "vaccinium macrocarpon" OR "vaccinium myrtillus" OR "vachellia farnesiana" OR "vachellia nilotica" OR "vachellia tortilis" OR valerian OR valeriana OR veratrum OR verbascum OR vinca OR "viscum album" OR vitex OR vitis OR voacanga OR "wild cherry" OR "wild yam" OR willow OR "witch hazel" OR withania OR woodfordia OR "wrightia tinctoria" OR "wrightia tomentosa" OR xanthium OR xanthorrhiza OR yarrow OR yew OR yohimbe OR yucca OR zanthoxylum OR "zataria multiflora" OR "zea mays" OR "zingiber officinale")) \| 143,436 \| \| S16 \| S14 OR S15 \| 191,199 \| \| S17 \| S10 OR S13 OR S16 \| 266,99 \| \| S18 \| (MH "Trigeminal Neuralgia") \| 1,404 \| \| S19 \| (MH "Trigeminal Nerve Diseases") \| 265 \| \| S20 \| TI ( ("drigeminal neuropath*" OR "epileptiform neuralg*" OR "fothergill disease*" OR prosopalgia OR "tic doloureux" OR "tic douloureux" OR "trifacial neuralg*" OR "trigemin* neuralg*" OR "trigemin* nerve disease*" OR "trigemin* nerve neuralgia" OR "trigemin* nerve disorder*" OR "trigemin* neuropathic pain" ) ) OR AB ( ("drigeminal neuropath*" OR "epileptiform neuralg*" OR "fothergill disease*" OR prosopalgia OR "tic doloureux" OR "tic douloureux" OR "trifacial neuralg*" OR "trigemin* neuralg*" OR "trigemin* nerve disease*" OR "trigemin* nerve neuralgia" OR "trigemin* nerve disorder*" OR "trigemin* neuropathic pain" ) ) \| 1,436 \| \| S21 \| TI ( (("trigemin* nerve" OR "mandibul* nerve" OR "mandibul* neuropathic" OR "maxilla* nerve" OR "chronic neuralgia" ) AND pain ) ) OR AB ( (("trigemin* nerve" OR "mandibul* nerve" OR "mandibul* neuropathic" OR "maxilla* nerve" OR "chronic neuralgia" ) AND pain ) ) \| 506 \| \| S22 \| S18 OR S19 OR S20 OR S21 \| 2,369 \| \| S23 \| S17 AND S22 \| 272 \| | |
